# Exploring the Relationship Between Obesity, Weight Loss and Health‐Related Quality of Life: An Updated Systematic Review of Reviews

**DOI:** 10.1111/cob.70049

**Published:** 2025-11-03

**Authors:** Tone Nygaard Flølo, Hui‐Hsuan Liu, John Roger Andersen, Ronette L. Kolotkin

**Affiliations:** ^1^ Department of Nursing and Health Promotion Oslo Metropolitan University Oslo Norway; ^2^ Department of Surgery Voss Hospital, Haukeland University Hospital Voss Norway; ^3^ Real‐World Evidence, OPEN Health Communications London UK; ^4^ Faculty of Health and Social Sciences Western Norway University of Applied Sciences Førde Norway; ^5^ Center of Health Research Førde Hospital Trust Førde Norway; ^6^ Duke Family Medicine and Community Health Duke University School of Medicine Durham North Carolina USA; ^7^ Western Norway University of Applied Sciences Førde Norway; ^8^ Morbid Obesity Centre Vestfold Hospital Trust Tønsberg Norway; ^9^ Quality of Life Consulting, PLLC Durham North Carolina USA

**Keywords:** health‐related quality of life, obesity, weight loss, weight management

## Abstract

The aim of this systematic literature review (SLR) of reviews was to update the evidence, established in a 2017 SLR, on the impact of obesity and weight loss by various interventions on health‐related quality of life (HRQoL). In total, eight SLRs and/or meta‐analyses published since 2017 were identified. The results consistently demonstrated a negative association between obesity and HRQoL, with some evidence suggesting poorer HRQoL in people with adverse metabolic profiles. In addition to substantial weight or body mass index reduction, metabolic and bariatric surgery (MBS) or endoscopic bariatric therapies resulted in significant and clinically relevant HRQoL improvements, with pronounced and persistent effects on the physical components in particular. HRQoL typically improved within 1–2 years after MBS and stabilised or deteriorated while remaining above baseline in subsequent years, likely due to weight regain. Evidence for the benefits of exercise interventions on HRQoL was inconclusive. Notably, no SLRs on anti‐obesity medications were identified, limiting conclusions on this emerging treatment area. This updated SLR expands on previous findings from the 2017 SLR, providing additional insights into underexplored areas, including the role of metabolic profiles in HRQoL and trends of HRQoL after MBS. Evidence synthesis remains challenging due to heterogeneity in HRQoL measurements used in this field.

**Summary**

- What is already known about this subject
  ○ Obesity has a significant negative impact on health‐related quality of life (HRQoL) in adults, with the most pronounced deficits seen for physical health domains.
  ○ Weight loss achieved through metabolic and bariatric surgery (MBS) is associated with substantial improvements in HRQoL.
  ○ By contrast, evidence of HRQoL benefits from non‐surgical lifestyle interventions is inconsistent and inconclusive, potentially due to historically lower weight reduction as compared to MBS.
- What this study adds
  ○ Adults with adverse metabolic profiles in addition to obesity tend to report poorer HRQoL outcomes than those with metabolically healthier phenotypes.
  ○ HRQoL improves significantly within 1–2 years after MBS, especially in the physical aspects, but often plateaus or declines over time, highlighting the need for long‐term follow‐up.
  ○ The methodological quality of systematic reviews on obesity and HRQoL needs improvement, particularly in research questions, protocol registration, search strategies, evidence grading and reporting standards.

**Abbreviations**
AMSTAR‐2: A Measurement Tool to Assess Systematic Reviews version 2
AOM: anti‐obesity medication
BMI: body mass index
BPD‐DS: biliopancreatic diversion with duodenal switch
CCA: corrected covered area
CCT: controlled clinical trial
CI: confidence interval
COOP: Dartmouth COOP Functional Health Assessment Charts
EBT: endoscopic bariatric therapy
EWL: excess weight loss
GIQLI: Gastrointestinal Quality of Life Index
HRQoL: health‐related quality of life
IGB: intragastric balloon placement
IWQOL: Impact of Weight on Quality of Life
LQ: Laval Questionnaire
MBS: metabolic and bariatric surgery
MCID: minimal clinically important difference
MCS: mental component score
ORC: obesity‐related complication
OWLQOL: Obesity and Weight Loss Quality of Life
PCS: physical component score
PICOS: Population, Intervention, Comparison, Outcomes and Study design
PRISMA: Preferred Reporting Items for Systematic Reviews and Meta‐Analyses
PRO: patient‐reported outcome
QOLOD: Quality of Life, Obesity and Dietetics Questionnaire
RAND‐36: RAND 36‐Item Health Survey
RCT: randomised controlled trial
RoB: risk of bias
SF‐12: 12‐Item Short Form Survey
SF‐36: 36‐Item Short Form Health Survey
SLR: systematic literature review
SMD: standardised mean difference
SOS: Swedish Obese Subjects
WHOQOL: World Health Organization Quality‐of‐Life Scale
WRSM: Weight‐Related Symptom Measure

## 1 Introduction

The global prevalence of obesity (defined as body mass index [BMI] ≥ 30.0 kg/m^2^) is rising. It is estimated that by 2035, 1.53 billion adults will be living with obesity, up from 0.81 billion in 2020 [1].

Health‐related quality of life (HRQoL) is a multidimensional measurement of self‐perceived health status, encompassing broad aspects of an individual's well‐being (e.g., physical, psychological and social functioning) [2]. The impact of obesity and/or weight loss on HRQoL is measured through generic and disease‐specific questionnaires [3]. Generic measures assess general aspects of HRQoL, whereas disease‐specific measures determine HRQoL associated with specific medical conditions or clinical populations. HRQoL measures are increasingly included in obesity research to inform patient‐centred clinical decision‐making and health policy [4].

The growing body of relevant literature reviews gave rise in 2017 to a systematic literature review (SLR) of reviews [3], which provided a comprehensive examination of the impact of obesity and weight loss on HRQoL in adults. The findings confirmed that obesity was associated with significantly reduced generic and obesity‐specific HRQoL. Weight loss through metabolic and bariatric surgery (MBS) consistently improved HRQoL, likely due to a greater weight reduction than with other available treatments. The effect of non‐surgical weight loss interventions on HRQoL showed mixed results, even in randomised controlled trials (RCTs). The review highlighted gaps in our understanding of factors contributing to variability in HRQoL outcomes.

Since 2017, the therapeutic landscape in obesity management has evolved, with advancements in pharmacotherapy and surgical techniques [5, 6, 7], as well as emerging digital health interventions [8]. Alongside lifestyle and behavioural therapy, anti‐obesity medications (AOMs) are recommended for adults with a BMI ≥ 30.0 kg/m^2^ or > 27.0 kg/m^2^ with obesity‐related complications (ORCs) [9, 10]. Across international guidelines, MBS is generally recommended for adults with a BMI ≥ 40.0 kg/m^2^ or ≥ 35.0 kg/m^2^ with ORCs [9, 10]. In 2023, the American Society for Metabolic and Bariatric Surgery/International Federation for the Surgery of Obesity and Metabolic Disorders published guidelines recommending that healthcare providers should consider offering MBS for individuals with a BMI ≥ 35.0 kg/m^2^ regardless of ORCs or ≥ 30.0 kg/m^2^ with ORCs (27.5 kg/m^2^ for Asian populations) [6].

Considering the expanding treatment landscape for obesity management, there is a need to assess whether new evidence meaningfully impacts the findings and conclusions established in the 2017 SLR [3]. The aim of this SLR was to evaluate the latest data on the impact of obesity and weight loss by different interventions on HRQoL.

## 2 Methods

### 2.1 Protocol and Registration

This SLR of reviews was undertaken in accordance with the Preferred Reporting Items for Systematic reviews and Meta‐Analyses (PRISMA) guidelines [11] and the Cochrane Handbook for Systematic Reviews of Interventions [12]. The protocol was registered in PROSPERO (CRD42024596534) before the search.

### 2.2 Eligibility Criteria

The inclusion and exclusion criteria specified in Table S1 were defined according to the Population, Intervention, Comparison, Outcomes and Study design (PICOS) framework [13]. To be eligible, studies had to be SLRs and/or meta‐analyses evaluating the impact of obesity and/or weight management on HRQoL; conducted in adults aged ≥ 18 years with obesity (defined as BMI ≥ 30.0 kg/m^2^); and written in English and published in peer‐reviewed journals.

### 2.3 Search Strategy

For this current search strategy, the 2017 SLR PubMed and Embase search was repeated to capture articles published from 1 January 2001 until 15 April 2025, and included three extra databases (MEDLINE, PsycInfo and CINAHL) plus additional search terms to capture a holistic overview of the current therapeutic landscape in obesity management. The applied search strings and results are detailed in Tables S2–S4.

Grey literature was searched in sources such as PROSPERO [14] to retrieve relevant articles not identified from database searches. A snowballing approach was undertaken in parallel to identify additional reviews from the reference list of retained articles.

Full citations for all retrieved references were imported into EndNote for storage, management and removal of duplicates.

### 2.4 Study Selection

**FIGURE 1:**
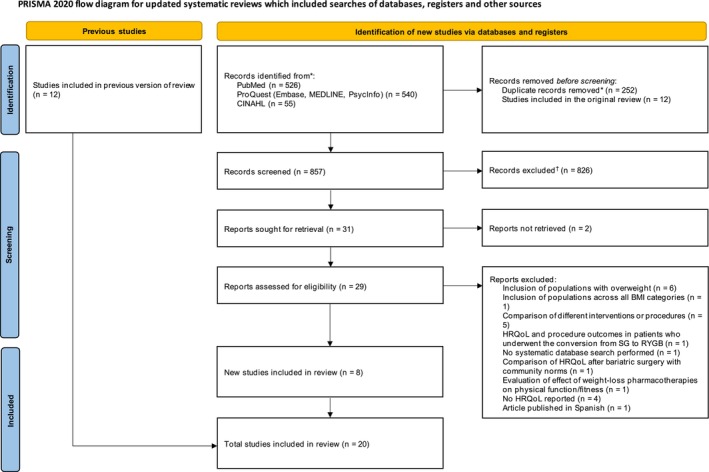
Preferred reporting items for systematic reviews and meta‐analyses (PRISMA) flow chart. The second reviewer performed title or abstract screening for 40% of overlaps (including 29 articles selected for full‐text screening). Disagreements between authors or reviewers on two of the 29 full‐text articles screened [15, 16] were resolved through consensus between the reviewers or with authors. *Systematic search results were deduplicated with EndNote before title or abstract screening. ^†^Including 25 duplicate records. BMI, body mass index; HRQoL, health‐related quality of life; RCT, randomised controlled trial; RYGB, Roux‐en‐Y gastric bypass; SG, sleeve gastrectomy; SLR, systematic literature review.

Titles and abstracts retrieved from the electronic database search were screened independently by two reviewers (Hui‐Hsuan Liu [HHL] and Olivia Rogerson [OR] of OPEN Health) for inclusion, using a pre‐piloted Excel spreadsheet comprising PICOS criteria. Discrepancies were resolved through consensus between the reviewers or with a third reviewer. The same process was applied to articles selected for full‐text review. Excluded articles were documented with reasons for exclusion according to the pre‐defined criteria (Table S1 and Figure 1). Authors manually checked reference lists from eligible studies to ensure inclusion of all potentially relevant studies. A complete list of excluded reviews with reasons for exclusion is detailed in Table S5.

During study selection, the eligibility criteria (Table S1) were strictly applied as defined in the registered protocol; in particular, only reviews focusing on obesity (defined as BMI ≥ 30.0 kg/m^2^) were selected for inclusion, whereas those evaluating the impact of overweight were excluded.

### 2.5 Data Extraction

As in the 2017 SLR [3], data from included reviews were extracted using a standardised, pre‐piloted form, with the following key variables: study details (e.g., type and goal of review, publication year), number and details of studies included in each review article (e.g., study design, study cohort, follow‐up period), key findings (e.g., intervention details, changes in HRQoL outcomes) and strengths and limitations of each review.

Data were extracted by one reviewer (HHL) and checked by a second reviewer (OR) for accuracy and completeness. Discrepancies were resolved through consensus between the reviewers or with a third reviewer. Missing data deemed important by the reviewers were requested from the corresponding authors of the original published reviews.

### 2.6 Methodological Quality Appraisal, Credibility of Evidence and Overlapping Citations

Confidence in the methodological quality of each review was determined using the A Measurement Tool to Assess Systematic Reviews version 2 (AMSTAR‐2) checklist, with a range from ‘high’ to ‘critically low’ (Table 1) [25]. Credibility of evidence was assessed using grading criteria proposed by Fusar‐Poli and Radua [26] (Table 2).

**TABLE 1:** Methodological quality assessed by AMSTAR‐2.

Reference (author, year)	AMSTAR‐2 checklist																	
	Item 1	Item 2	Item 3	Item 4	Item 5	Item 6	Item 7	Item 8	Item 9	Item 10	Item 11	Item 12	Item 13	Item 14	Item 15	Item 16	Overall confidence	
Jayedi et al., 2024 [17]	Y	Y	Y	Y	Y	Y	Y	Y	Y	N	Y	Y	Y	Y	Y	Y	H	Item 1. Did the research questions and inclusion criteria for the review include the components of PICO? Item 2. Did the report of the review contain an explicit statement that the review methods were established prior to the conduct of the review and did the report justify any significant deviations from the protocol? Item 3. Did the review authors explain their selection of the study designs for inclusion in the review? Item 4. Did the review authors use a comprehensive literature search strategy? Item 5. Did the review authors perform study selection in duplicate? Item 6. Did the review authors perform data extraction in duplicate? Item 7. Did the review authors provide a list of excluded studies and justify the exclusions? Item 8. Did the review authors describe the included studies in adequate detail? Item 9. Did the review authors use a satisfactory technique for assessing the RoB in individual studies that were included in the review? Item 10. Did the review authors report on the sources of funding for the studies included in the review? Item 11. If meta‐analysis was performed did the review authors use appropriate methods for statistical combination of results? Item 12. If meta‐analysis was performed, did the review authors assess the potential impact of RoB in individual studies on the results of the meta‐analysis or other evidence synthesis?
Nakanishi et al., 2023 [18]	Y	Y	N	PY	Y	Y	N	Y	Y	N	Y	N	Y	Y	Y	Y	CL	
Vega‐Albornoz et al., 2023 [19]	Y	N	N	PY	Y	Y	N	Y	N	N	Y	N	N	Y	Y	Y	CL	
Abiri et al., 2022 [20]	Y	N	N	Y	Y	Y	N	PY	Y	N	NM	NM	N	Y	NM	Y	CL	
Sierżantowicz et al., 2022 [21]	Y	Y	N	PY	Y	Y	N	Y	Y	N	NM	NM	Y	N	NM	Y	CL	
Gadd et al., 2020 [22]	Y	Y	N	Y	Y	Y	N	Y	Y	Y	Y	Y	Y	Y	N	Y	CL	
Baillot et al., 2018 [23]	Y	Y	Y	PY	Y	Y	N	Y	Y	Y	Y	Y	Y	Y	Y	Y	L	
Raaijmakers et al., 2017 [24]	Y	N	Y	PY	Y	Y	N	PY	Y	N	NM	NM	Y	Y	NM	Y	CL	
																		Item 13. Did the review authors account for RoB in individual studies when interpreting/discussing the results of the review? Item 14. Did the review authors provide a satisfactory explanation for, and discussion of, any heterogeneity observed in the results of the review? Item 15. If they performed quantitative synthesis did the review authors carry out an adequate investigation of publication bias (small study bias) and discuss its likely impact on the results of the review? Item 16. Did the review authors report any potential sources of conflict of interest, including any funding they received for conducting the review?

Abbreviations: AMSTAR‐2, A Measurement Tool to Assess Systematic Reviews version 2; PICO, Population, Intervention, Comparison, Outcomes; RoB, risk of bias.

Checklist options: 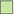, yes; 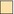, partial yes; 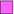, no; 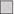, no meta‐analysis conducted.

Overall confidence rating: 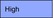, ≤ 1 non‐critical weaknesses; 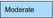, ≥ 2 non‐critical weaknesses, with no critical weaknesses; 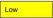, 1 critical weakness, with or without non‐critical weaknesses; 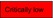, ≥ 2 critical weaknesses, with or without non‐critical weaknesses.

**TABLE 2:** Credibility of evidence.

Reference (author, year)	Credibility of evidence [26]									
	Meta‐analysis conducted	Number of cases (> 1000)	*p* < 10^−6^	*p* < 10^−3^	*p* < 0.05	*p* > 0.05	*I* ^2^ < 50%	95% prediction interval excluding the null	No small‐study effects and no excess significance bias	Grading
Jayedi et al., 2024 [17]	N^a^	N/A	N/A	N/A	N/A	N/A	N/A	N/A	N/A	N/A
Nakanishi et al., 2023 [18]	Y	N	NT^b^	NT^b^	NT^b^	NT^b^	N	Y	N	N/A^b^
Vega‐Albornoz et al., 2023 [19]	Y	Y	N	N	Y	N	Y	Y	Y	Class II
Abiri et al., 2022 [20]	N	N/A	N/A	N/A	N/A	N/A	N/A	N/A	N/A	N/A
Sierżantowicz et al., 2022 [21]	N	N/A	N/A	N/A	N/A	N/A	N/A	N/A	N/A	N/A
Gadd et al., 2020 [22]	Y	N	N	Y	N	N	Y	Y	N	Class IV
Baillot et al., 2018 [23]	Y	N	N	N	N	Y	Y	N	N	Non‐significant
Raaijmakers et al., 2017 [24]	N	N/A	N/A	N/A	N/A	N/A	N/A	N/A	N/A	N/A

Abbreviations: HRQoL, health‐related quality of life; N/A, not applicable; NT, statistical significance not tested.

Checklist options: 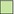, yes; 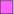, no; 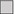, no meta‐analysis conducted.

Grading system: 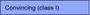, when number of cases is > 1000, *p* < 10^−6^, *I* ^2^ < 50%, 95% prediction interval excluding the null, no small‐study effects, and no excess significance bias; 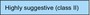, when number of cases is > 1000, *p* < 10^−6^, largest study with a statistically significant effect, and class I criteria not met; 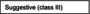, when number of cases is > 1000, *p* < 10^−3^, and class I and II criteria are not met; 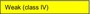, when *p* < 0.05 and class I–III criteria are not met; 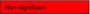, when *p* > 0.05.

^a^ No meta‐analysis was performed for HRQoL outcomes.

^b^ Mean differences in HRQoL between baseline and mid‐term follow‐up after metabolic and bariatric surgery were reported with reference to minimal clinically important differences; no statistical significance was tested.

Methodological quality appraisal and credibility of evidence were conducted by one reviewer (HHL) and validated by a second reviewer (OR) for accuracy for 20% of included articles. Discrepancies were resolved through consensus between the reviewers or with a third reviewer. The extent of overlap among studies included in the retained review articles was quantified to ensure non‐duplication of evidence, using the corrected covered area (CCA) formula [27]. A CCA value of 0–5 was considered as ‘slight overlap’, 6–10 as ‘moderate’, 11–15 as ‘high’ and > 15 as ‘very high’ [27].

### 2.7 Data Synthesis

A narrative synthesis was conducted; no statistical meta‐analyses of aggregated data were performed due to substantial heterogeneity across included reviews (e.g., interventions, follow‐up, HRQoL measures) with varied methodological quality. Results were tabulated and stratified by study characteristics for individual review articles, type or goals of review and key findings.

## 3 Results

### 3.1 Search Results

The database search yielded 1121 records with 31 articles full‐text screened for eligibility, of which 23 were excluded with justification according to the pre‐defined criteria (Table S5) and 8 were selected for inclusion [18, 19, 20, 21, 22, 23, 24]. The study selection process is presented in Figure 1. Table S6 provides an overview of the eight reviews meeting the inclusion criteria.

Key findings and evidence gaps are summarised in Table 3. Of these, three were SLRs [20, 21, 24], three were meta‐analyses [18, 19, 22] and two were SLRs and meta‐analyses [17, 23]. Only three reviews pre‐specified HRQoL as the primary outcome measure [18, 22, 24].

**TABLE 3:** Key findings from review articles.

Reference (author, date)	Type/goal of review	Studies	Contribution of new evidence	Limitations/gaps
Category 1: Relationship between weight/BMI and HRQoL (baseline/preintervention)				
Abiri et al., 2022 [20]	An SLR to evaluate the association between different obesity phenotypes with common psychiatric symptoms and HRQoL	18 studies: longitudinal studies (*n* = 4) and cross‐sectional studies (*n* = 14) 2008–2021	This is the first SLR to provides a novel perspective on the impact of metabolic profile on HRQoL in adults with obesity While obesity is strongly associated with impaired HRQoL, HRQoL is worse in the presence of an adverse metabolic profile (i.e., metabolic syndrome)	All included studies used generic or mental health‐specific measures No obesity‐specific measures were used No statistical modelling approach and mediation tests were implemented to explore the link between different obesity phenotypes and HRQoL outcomes
Category 2: HRQoL after exercise intervention in adults with obesity				
Baillot et al., 2018 [23]	An SLR and MA to evaluate the effect of exercise on psychosocial outcomes in adults with obesity Comparisons of exercise group vs. no exercise control group, or before vs. after exercise	22 studies (across 23 articles): RCTs (*n* = 16), CCT (*n* = 1) and nonrandomised pre–post studies (*n* = 5) Duration of intervention ranged from 6 to 52 weeks, with most studies (*n* = 15) having < 16 weeks' follow‐up 2011–2017	This review contributes to the body of evidence in the original review suggesting that the impact of weight loss after lifestyle interventions on HRQoL remains insignificant, primarily due to high heterogeneity across available studies (study design, interventions), short‐term follow‐ups and low quality of evidence The findings may implicate the importance of using a disease‐specific HRQoL measure rather than generic measures to assess outcomes of interest	Generic HRQoL measures were most commonly used across studies (*n* = 12/22), whereas obesity‐specific measures were employed in a limited number of studies (*n* = 4/22) Data with long‐term follow‐up are limited
Category 3: HRQoL after weight loss (randomised controlled trials only)				
Jayedi et al., 2024 [17]	An SLR and MA to investigate the dose–response association of aerobic exercise of varying intensity with measures of body weight, waist size and fat mass in adults with overweight or obesity, with HRQoL prespecified as the secondary outcome measure	116 RCTs, with ≥ 8 weeks of intervention duration Intervention durations ranged from 8 to 64 weeks 1990–2023	Results from 1 RCT with 26 weeks of follow‐up showed that aerobic exercise training was associated with significant improvements in PCS and MCS of SF‐36 compared with the control group	No obesity‐specific measures were used Data from RCTs were very limited regarding the impact of weight loss through exercise interventions on HRQoL
Category 4: HRQoL after metabolic and bariatric procedure				
Raaijmakers et al., 2017 [24]	An SLR to evaluate short‐ and long‐term effects of MBS on HRQoL and comparison with community norms (i.e., general population samples)	40 prospective pre‐post studies, with ≥ 6 months' follow‐up Follow‐up ranged from 6 months to 10 years 2001–2016	The review provides insights into long‐term trends of HRQoL after MBS, with the maximum HRQoL increase reached after 1 year and the increase reaching a plateau phase in the subsequent years When comparing post‐MBS HRQoL with community norms, only outcomes assessed by obesity‐specific measures showed that HRQoL substantially improved and exceeded community norms The findings highlight the importance of using a more specific HRQoL measure than generic measures for the target populations or outcomes of interest	A limited number of studies used both generic and obesity‐specific measures (*n* = 4) As noted by the authors, PRO measures are highly variable and there are limited standards of study designs and reporting of HRQoL data in this area of research Data with long‐term follow‐up (≥ 10 years) post MBS are very limited
Gadd et al., 2020 [22]	An MA to examine the effect of EBTs on HRQoL and mental health of adult patients	20 studies (across 21 articles): observational studies (*n* = 15) and RCTs (*n* = 5) Follow‐up ranged from 4 to 76 months 2009–2018	This is the first MA that provides perspectives on the impact of EBTs on HRQoL Significant HRQoL improvements after EBTs, as assessed by generic and/or obesity‐specific measures, were consistently reported across studies The pooled estimates from MA showed that IGB was associated with significant HRQoL improvements	A limited number of studies used both generic and obesity‐specific measures (*n* = 2) Reporting of HRQoL data varied across studies The MA was limited by a small number of studies with sufficient data on pre‐ and post‐EBT HRQoL outcomes The interpretation of results from MA may not be extended to other types of EBT Data with long‐term follow‐up are very limited
Sierżantowicz et al., 2022 [21]	An SLR to evaluate HRQoL as an outcome measure in patients after MBS of any type and with ≥ 9 years' follow‐up	18 studies: nonrandomised clinical trials (*n* = 2), randomised clinical trials (*n* = 2), a mail survey (*n* = 1), prospective cohort studies (*n* = 6), observational studies (*n* = 2), a phone survey (*n* = 1), retrospective analyses (*n* = 3) and a prospective study (*n* = 1), with ≥ 9 years' follow‐up Follow‐up ranged from 0.5 to > 20 years 2015–2019	The review provides valuable insights into long‐term HRQoL trends post‐MBS, which is an area not commonly addressed in existing research HRQoL began to decrease within the initial 5–6 years post‐MBS and stabilised up to 9–12 years while remaining above the baseline values, suggesting a persistent beneficial effect of MBS on quality of life	Generic measure SF‐36 was the most commonly used (alone or with other measures) for assessing HRQoL (*n* = 9/14), whereas obesity‐specific measures were employed in a limited number of studies (*n* = 5/14) A limited number of studies used both generic and obesity‐specific measures (*n* = 4) Data with long‐term follow‐up (≥ 10 years) post MBS are sparce
Nakanishi et al., 2023 [18]	An MA to assess the impact of BPD‐DS on mid‐term HRQoL in the management of obesity	12 studies, including retrospective cohort studies (*n* = 6) and prospective cohort studies (*n* = 6) Mean follow‐up ranged from 2.0–12.3 years English only 2005–2022	This is the first MA that evaluates the effect of BPD‐DS on HRQoL with reference to MCID^a^ The findings provide valuable insights into the clinical relevance of BPD‐DS and the role of such surgical procedure in the management of obesity HRQoL improvements, as assessed by generic and/or obesity‐specific measures achieved MCID after BPD‐DS	Generic measure SF‐36 was most commonly used across studies (*n* = 7/12), whereas obesity‐specific measures were employed in a limited numbers of studies (*n* = 7/12) A limited number of studies used both generic and obesity‐specific measures (*n* = 4) Data with long‐term follow‐up are very limited No statistical modelling approach and mediation tests were implemented to explore the link between weight/BMI reduction and improved HRQoL
Vega‐Albornoz et al., 2023 [19]	An MA to determine the effect of MBS on HRQoL based on the dimensions of social, mental, physical and emotional function in patients with obesity	Three SLRs/MA (of 25 clinical trials) Follow‐up ranged from 3 months to 10 years 2016–2022	The results of this MA of a large sample size strengthen the findings established in the original review that HRQoL significantly improved within 1 year after MBS in patients with obesity The short‐term effect of MBS on HRQoL was consistently reported in the literature, with the largest improvement observed in the physical function vs. other HRQoL domains	Only SF‐36 data were analysed No statistical modelling approach and mediation tests were implemented to explore the link between weight/BMI reduction and improved HRQoL Data with long‐term follow‐up are very limited

Abbreviations: BMI, body mass index; BPD‐DS, biliopancreatic diversion with duodenal switch; CCT, controlled clinical trial; EBT, endoscopic bariatric therapy; HRQoL, health‐related quality of life; IGB, intragastric balloon placement; MA, meta‐analysis; MBS, metabolic and bariatric surgery; MCID, minimal clinically important difference; PRO, patient‐reported outcome; SF‐36, 36‐Item Short Form Health Survey; SLR, systematic literature review.

^a^ MCID was defined as a five‐point increase in PCS or MCS of SF‐36. The range of MCID for LQ domains is 0.6–2.0 (symptoms: 0.8; activity/mobility: 0.9; personal hygiene/clothing: 1.4; emotions: 1.2; social interactions: 1.2; and sexual life: 2.0). The range of MCID for the IWQOL‐Lite total score is 7.7–12.0 points (depending on baseline severity).

In the eight included reviews, a total of 134 individual studies were evaluated, with only two overlapping studies identified (Table S7). The CCA value of 0.0021 indicates a slight level of overlap across reviews.

The included reviews are described below by category as organised in the 2017 SLR [3].

### 3.2 Evidence Quality

The overall confidence in methodological quality was rated ‘high’ for one review [17] and ‘low’ [23] to ‘critically low’ [18, 19, 20, 21, 22, 24] for the remaining seven reviews (Table 1). Three of the four reviews with meta‐analyses performed for HRQoL outcomes were assessed for credibility of evidence, with one [19] graded as ‘highly suggestive’, one [22] considered ‘weak’ and one [23] rated as ‘non‐significant’ (Table 2).

### 3.3 Findings

#### 3.3.1 Assessment of Health‐Related Quality of Life

Among the eight reviews assessed, 36 different HRQoL measures were identified, including eight generic, eight obesity‐specific, one bariatric surgery‐specific, two gastrointestinal‐specific, two combined generic and obesity‐specific, one combined obesity‐specific and bariatric surgery‐specific, five other disease‐related and nine mental health‐specific measures (Table S8).

Of the eight reviews, six included studies that reported HRQoL outcomes measured by validated instruments of any type [18, 20, 21, 22, 23, 24], whereas two were restricted to studies reporting HRQoL with the 36‐Item Short Form Health Survey (SF‐36) [17, 19]. Across all studies included in the eight reviews, the most commonly used HRQoL measures were the generic SF‐36/RAND 36‐Item Health Survey (RAND‐36) and the obesity‐specific Impact of Weight on Quality of Life (IWQOL)‐Lite [28, 29, 30]. Four reviews comprised studies that used both generic and obesity‐specific measures: four studies [31, 32, 33, 34] included in the review by Sierżantowicz et al. [21], four [35, 36, 37, 38] in the review by Nakanishi et al. [18], two [39, 40] in the review by Gadd et al. [22] and four [41, 42, 43, 44] in the review by Raaijmakers et al. [24].

As with the 2017 SLR [3], results of other disease‐related measures, gastrointestinal‐specific measures and measures of HRQoL surrogate markers (i.e., depression, anxiety, stress or emotions) were not reported as these are not true assessments of HRQoL. Results from bariatric surgery‐specific measures (i.e., Bariatric Analysis and Reporting Outcome System) were also excluded due to their relevance for post‐surgical outcomes only [45].

#### 3.3.2 Category 1: Relationship Between Weight/Body Mass Index and Health‐Related Quality of Life (Baseline/Pre‐Intervention)

One review [20] evaluated the association between different obesity phenotypes with common psychiatric symptoms and HRQoL, without any interventions. Across 18 primary studies (*n* = 14 cross‐sectional, *n* = 4 longitudinal), three obesity phenotypes were investigated: metabolically healthy individuals with obesity, metabolically abnormal individuals with healthy weight and metabolically unhealthy individuals with obesity [20]. HRQoL was assessed in 7 of the 18 included studies using three different validated generic measures: SF‐36, 12‐Item Short Form Survey (SF‐12) and EuroQol‐5 Dimension. The results demonstrated that obesity was strongly associated with impaired HRQoL, which was further impaired in the presence of adverse metabolic profiles (i.e., metabolic syndrome).

#### 3.3.3 Category 2: Health‐Related Quality of Life After Exercise Intervention in Adults With Obesity

Baillot et al. [23] performed an SLR and meta‐analysis to evaluate the effect of exercise on psychosocial outcomes in adults with obesity across 22 primary studies with mixed designs. Twenty of the 22 included studies examined overall HRQoL, including 14 RCTs, one controlled clinical trial (CCT) and five non‐randomised pre–post studies. Of the 14 RCTs, 10 used generic HRQoL measures (SF‐12 or SF‐36), one used an obesity‐specific measure (IWQOL‐Lite) and five used other disease‐related measures. The CCT used a generic HRQoL measure (World Health Organization Quality‐of‐Life Scale [WHOQOL]). Of the five pre–post studies, three used obesity‐specific measures (IWQOL‐Lite and Quality of Life, Obesity and Dietetics Questionnaire [QOLOD]) and one used a generic HRQoL measure (Dartmouth Primary Care Cooperative Research Network and the World Organization of National Colleges, Academies and Academic Associations of General Practitioners/Family Physicians [COOP/WONCA]), whereas another used Gordon's Functional Pattern Assessment, which is not considered a generic HRQoL measure in the current review (Table S9), despite being considered valid by Baillot et al. [23].

In the 10 RCTs using SF‐12 or SF‐36, most studies (*n* = 6) showed no significant differences in HRQoL changes between the exercise and control groups; however, one reported significant HRQoL improvements in the exercise group relative to pre‐intervention (but with no significant difference vs. control group), one reported significant differences in change in physical component score (PCS) (but not mental component score [MCS]) between groups favouring the exercise group, and two reported significant differences in change in several physical and mental HRQoL subscales between groups favouring the exercise group. The CCT, using a generic measure (WHOQOL), showed a significant between‐group interaction favouring the exercise group. Among the five pre‐post studies, three using obesity‐specific measures (IWQOL‐Lite, QOLOD) and one using a generic measure (COOP/WONCA), all reported statistically significant improvements in total HRQoL scores and/or several domain scores (e.g., psychosocial functioning, physical functioning, self‐esteem, public distress), albeit based on limited sample sizes (*n* = 8–25).

In the meta‐analysis comprising seven RCTs that assessed HRQoL, no statistically significant effect favouring exercise was detected for either physical or mental components of HRQoL as assessed by SF‐36 or SF‐12 in adults with obesity, despite low heterogeneity across studies. Sensitivity analyses were performed to ensure there was no effect of diet combined with exercise or control groups on the outcome measures between the exercise and control groups. Overall, the authors interpreted the results taking into account potential publication bias and low quality of evidence from available studies [23].

Currently, data are limited to support the benefits of exercise interventions on HRQoL in adults with obesity.

#### 3.3.4 Category 3: Health‐Related Quality of Life After Weight Loss (Randomised Controlled Trials Only)

One review and meta‐analysis of 116 RCTs investigated the efficacy of aerobic exercises in weight‐loss management among individuals with overweight or obesity, with HRQoL pre‐specified as the secondary outcome measure [17]. The primary analyses yielded an inverse dose–response association of aerobic exercise at moderate intensity or higher with body weight, waist size and body fat. Yet, meta‐analyses on HRQoL were restricted due to a limited number of trials reporting HRQoL outcomes. Results based on one RCT [46] demonstrated an association between aerobic exercise training and improved PCS and MCS of SF‐36 among older adults with obesity. Effect sizes showed a relatively greater increase in PCS than MCS (Table S6). However, conclusions were limited by low credibility of evidence.

#### 3.3.5 Category 4: Health‐Related Quality of Life After Metabolic and Bariatric Procedure

Five reviews examined HRQoL after MBS or endoscopic bariatric therapy (EBT). There was one overlapping primary study between reviews by Sierżantowicz et al. [21] and Raaijmakers et al. [24]; the remaining three reviews [18, 19, 21] had no overlapping studies (Table S7).

Three of five reviews reported significant weight or BMI reduction following MBS or EBT, with variations in methods used for analysis and reporting across and within reviews [18, 21, 22]. In the review by Sierżantowicz et al. with ≥ 9 years' follow‐up after MBS [21], weight loss was expressed as mean excess weight loss (EWL) ranging from 10.0% to 63.4%, or as mean change in BMI ranging from 46.5 to 51.9 kg/m^2^ at baseline (pre‐operation) to 32.0 to39.4 kg/m^2^ at follow‐up. In the review by Gadd et al. [22], mean EWL after EBT ranged from 17.6% to 56.0%, mean weight loss ranged from 6.5 to 25.6 kg and mean BMI reduction ranged from 3.9 to 7.8 kg/m^2^, estimated across studies with 4–76 months of follow‐up. Nakanishi et al. [18] estimated an absolute BMI reduction of 22.6 kg/m^2^, a 68.9% excess BMI reduction and an 86.3% EWL over a mean follow‐up of 2.0–12.3 years after MBS. Two reviews did not report changes in weight or BMI [19, 24].

HRQoL outcome analyses and data format varied widely, limiting their comparability. Sierżantowicz et al. [21] and Raaijmakers et al. [24] performed narrative syntheses, whereas Vega‐Albornoz et al. [19], Nakanishi et al. [18] and Gadd et al. [22] reported estimated effect sizes for HRQoL changes. Alongside various HRQoL instruments, HRQoL data reporting differed, with studies presenting total, composite or different subscale scores. This heterogeneity in methodologies and HRQoL data presentation within each review restricts meaningful comparisons across reviews.

##### 3.3.5.1 SF‐36 Results of Bariatric Procedure Studies

Vega‐Albornoz et al. [19] performed a meta‐analysis to determine the effect of MBS on HRQoL, focusing on SF‐36 dimensions of social, mental, physical and emotional function. The meta‐analysis included three SLRs and/or meta‐analyses that evaluated five types of MBS during follow‐up periods of 3–12 months after surgery. Pooled estimates showed significant improvements in total HRQoL scores and domain scores after MBS, albeit with considerable heterogeneity across studies [19]. Notably, the weighted mean effect size was relatively larger in physical function scores than in other domain scores (i.e., social, mental and emotional function) [19]. Meta‐regression further confirmed no effects of follow‐up time (3–6 months vs. 6–12 months) on the overall findings [19]. The authors speculated that the variability of the results was mostly linked to heterogeneity, given an absence of publication bias. However, no assessment of the risk of bias (RoB), methodological quality and credibility of evidence was performed, thus limiting the interpretation of the results [19].

Sierżantowicz et al. [21] performed an SLR to evaluate HRQoL in patients undergoing MBS of any type in studies with ≥ 9 years' follow‐up. The review comprised 18 studies with various study designs and follow‐up durations ranging from 0.5 to > 20 years. Of the 10 studies using SF‐36, 4 showed that HRQoL began to decrease within 5–6 years after MBS, but scores at 9–12 years did not significantly differ from scores at 5 years. Six of these 10 studies demonstrated that HRQoL scores at 9–12 years remained significantly higher than baseline levels despite a decrease from previous years. Furthermore, three studies demonstrated a negative correlation between HRQoL and weight or BMI [31, 34, 35]. Notably, 4/18 studies reported trends suggesting improvements in PCS and MCS of SF‐36 after MBS, but results were inconsistent. Three of these four studies demonstrated that MCS appeared to deteriorate faster than PCS and returned to baseline levels at the end of follow‐up (6–12 or 9–10 years). However, different results were observed in one study [47], that is, significant improvements in MCS were observed at 2 years after MBS and persisted at 10 years, but this was not the case for PCS. Overall, the authors acknowledged the potential RoB that may hinder interpretation of the results [21].

Nakanishi et al. [18] performed a meta‐analysis based on 12 cohort studies (*n* = 6 retrospective, *n* = 6 prospective) to assess the impact of biliopancreatic diversion with duodenal switch (BPD‐DS) on mid‐term HRQoL. Of note, the definition of ‘mid‐term’ was not specified. The pooled estimates derived from seven studies showed that minimal clinically important differences (MCIDs) were achieved for PCS but not MCS (Table S6). All SF‐36 subscales also achieved MCIDs, with physical function demonstrating the largest mean increase (Table S6). Despite positive findings from the meta‐analysis, data should be interpreted with caution given the limited sample size and small number of studies [18].

Raaijmakers et al. [24] conducted an SLR to evaluate the short‐ and long‐term effects of MBS on HRQoL and to compare results with community norms (defined as general population samples). Forty prospective studies with pre‐post study design that assessed MBS of any type were included, with follow‐up between 6 months and 10 years. Fourteen different HRQoL measures were used across studies, with SF‐36/RAND‐36 being the most common (*n* = 26/40). All included studies reported a significant HRQoL increase after MBS (all *p* ≤ 0.05) [24], and most studies showed that significant HRQoL improvements occurred 1 year after MBS and stabilised in subsequent years. Notably, a greater magnitude of improvement was observed for PCS than MCS across all 25 studies with available data, indicating that MBS has a greater impact on physical components of SF‐36/RAND‐36 than mental components [24]. All 25 studies with available data showed improvement in PCS of SF‐36/RAND‐36 following MBS [24]. Improvement in MCS of SF‐36/RAND‐36 was also observed in nearly all studies (*n* = 22/25), although three studies reported no evidence of improvements in MCS within 1 year after MBS [24].

When comparing post‐MBS HRQoL with community norms, results were inconsistent across studies [24]. Of the 25 studies using SF‐36/RAND‐36, 15 showed that post‐operative PCS exceeded community norms, but the remaining 10 studies demonstrated post‐operative PCS below community norms. Similarly, 12 of the 25 studies reported post‐operative MCS exceeded community norms, whereas 13 showed post‐operative MCS below community norms [24].

In the meta‐analysis, the pooled summary scores were calculated for SF‐36 and compared with community norms [24]. The pooled summary scores of post‐operative PCS and MCS not only increased compared with pre‐operative but also exceeded the community norms (Table S6) [24].

Most included studies were determined to be of ‘fair’ to ‘good’ methodological quality (*n* = 35/40) [24]. Although results were considered to be reliable and generalisable to the target population, given the inclusion of only prospective studies and a relatively large sample size (*N* = 7720), the authors did not rule out the possibility of publication bias and selection bias [24].

Gadd et al. [22] performed a meta‐analysis to examine the effect of EBTs on HRQoL and mental health in adults with obesity. EBTs are new, minimally invasive, non‐surgical bariatric procedures that mimic the anatomical alterations created by MBS through selectively targeting the stomach or the small intestines using specific endoscopically placed devices [48, 49]. Twenty studies (across 21 articles) evaluating five different EBTs were included in the meta‐analysis, comprising 15 observational studies and five RCTs [22]. All studies used a range of HRQoL measures (except one that used a single generic measure), with two studies [39, 40] using a generic measure as well as an obesity‐specific measure [22].

In the qualitative synthesis, all eight studies using SF‐36 showed significant and consistent improvements in HRQoL from baseline to post‐EBT follow‐up [22]. However, changes in HRQoL outcomes were calculated based on available data across studies; that is, some were reported with mean changes in total score, whereas others were reported with different domain scores (e.g., general health, physical and/or mental functioning scores) [22]. Four studies reported significant improvements in mental health domain scores of SF‐36 after EBT [22].

In the meta‐analysis, pooled estimates from 8/20 studies showed that IGB was associated with significant HRQoL improvements (Table S6) [22]. However, publication bias was not assessed due to a small number of studies included in the meta‐analysis, and HRQoL outcomes assessed by different instruments (i.e., generic, obesity‐specific and gastrointestinal‐specific) were pooled in the analysis. Given the short follow‐up period and that the impact of other types of EBT on HRQoL was not assessed via meta‐analysis due to insufficient data, the findings and conclusions may not be generalisable to broader contexts [22].

Conclusions may be hindered by concerns surrounding overall RoB across 20 studies and ‘low’ to ‘very low’ certainty of evidence across studies reporting HRQoL and mental health due to RoB and observational study design [22]. Considering a number of study limitations, the authors concluded that EBT, particularly IGB, may improve HRQoL and mental health alongside weight loss; however, the long‐term effect on HRQoL and mental health remains unknown as most studies had short‐to‐intermediate follow‐ups (6–12 months) [22].

##### 3.3.5.2 Obesity‐Specific HRQoL Results of Bariatric Procedure Studies

In the review by Sierżantowicz et al. [21], five of 18 studies used obesity‐specific measures to assess the effect of MBS on HRQoL (Table S8). All studies demonstrated that HRQoL decreased within 5–6 years after MBS but stabilised at 9–12 years. Furthermore, three studies using Bariatric Quality of Life (*n* = 2) and IWQOL‐Lite (*n* = 1) showed that HRQoL improvement was significantly correlated with weight loss (or satisfaction with weight loss), and that HRQoL deterioration was associated with weight regain [21].

In the review by Nakanishi et al. [18], three of 12 studies using obesity‐specific measures reported clinically relevant HRQoL improvements at mid‐term follow‐up after BPD‐DS. In two studies, MCIDs were attained across all subscales of the Laval Questionnaire (LQ), with the largest mean difference observed in the activity/mobility subscale (Table S6). In one study, the mean difference in IWQOL‐Lite scores between the baseline and the mid‐term follow‐up period also achieved MCID. Moreover, two of 12 studies showed an increase in the summary score of the Obesity‐Related Problems Scale at the mid‐term follow‐up, but its clinical relevance was not determined (Table S6). Given the limited sample size and small number of studies, data should be interpreted cautiously [18].

Seven different obesity‐specific measures and two types of combined generic and obesity‐specific measures were used across 16 of the 40 studies reviewed by Raaijmakers et al. [24] (Table S8). Although all studies demonstrated significant HRQoL improvements after MBS, the results compared with community norms were inconsistent across eight studies with available data [24]. One study using IWQOL showed that post‐operative physical and mental health (i.e., psychosocial) composite scores were above community norms. Of five studies using IWQOL‐Lite, three demonstrated post‐operative total scores above community norms, whereas two showed scores below community norms [24]. In one study using Obesity and Weight Loss Quality of Life (OWLQOL) in conjunction with Weight‐Related Symptom Measure (WRSM), post‐operative OWLQOL total score was higher than community norms, whereas post‐operative WRSM total score remained below community norms [24]. In one study using the Swedish Obese Subjects (SOS) quality of life survey, all post‐operative domain scores were below community norms [24]. Taken together, most studies (*n* = 5/8) that employed IWQOL, IWQOL‐Lite or OWLQOL showed that post‐MBS HRQoL substantially improved and exceeded community norms [24].

In the review by Gadd et al. [22], all eight studies using IWQOL‐Lite (*n* = 6) or IWQOL (*n* = 2) demonstrated significant improvements in HRQoL and mental health domain scores after EBT alongside significant weight or BMI reduction. Changes in IWQOL and IWQOL‐Lite total scores were calculated for all studies, except for one study with only IWQOL‐Lite domain scores available [22].

## 4 Discussion

### 4.1 Summary of Evidence

Our findings not only reinforce the foundational conclusions of the 2017 SLR [3] but also provide critical new dimensions to our understanding of the association between obesity, weight loss and HRQoL.

The 2017 SLR established the negative impact of obesity on HRQoL and the consistent, positive effect of MBS, while noting the inconclusive evidence for non‐surgical interventions [3]. The present update confirms these core findings. For example, the meta‐analysis by Baillot et al. [23] echoes the earlier ambiguity surrounding the impact on HRQoL of weight loss after lifestyle interventions. However, where the 2017 SLR highlighted a scarcity of long‐term follow‐up data as a key limitation [3], the review by Sierżantowicz et al. [21] begins to fill this gap by providing HRQoL trajectories beyond 9 years after MBS [21]. Furthermore, we introduce evidence in areas not covered previously, such as the moderating role of metabolic phenotypes on HRQoL [20] and the emerging HRQoL outcomes associated with EBT [22].

Specifically, Abiri et al. [20] found that individuals with adverse metabolic profiles experienced poorer HRQoL compared with metabolically healthy individuals, although findings varied due to heterogeneous study designs, HRQoL measures and definitions of metabolic status [20]. Abiri et al. are the first to systematically examine the impact of obesity phenotypes on HRQoL, adding a novel perspective to this field [20]. Four reviews consistently demonstrated that MBS led to significant, short‐term HRQoL improvements in addition to clinically meaningful weight loss [19, 21, 22, 24]. Sierżantowicz et al. provided valuable data on long‐term (≥ 9 years) HRQoL trends [21], showing initial improvements at 1–2 years after MBS followed by deterioration at 5–6 years, yet HRQoL remained above baseline levels [21]. These long‐term HRQoL patterns were strongly correlated with weight or BMI changes, with initial HRQoL improvements linked to substantial weight loss and later declines associated with weight regain [21]. The strong correlation observed between HRQoL decline and weight regain after MBS likely reflects multifactorial mechanisms across biological, psychological and social domains [50, 51]. Biologically, weight regain may reintroduce physical burdens such as impaired mobility and increased bodily pain, which directly affect the physical functioning domains of instruments like the SF‐36. Psychologically, weight regain is often perceived as a personal failure, which may trigger symptoms of depression and anxiety, and declining self‐esteem or public distress captured by both generic measures and obesity‐specific measures such as the IWQOL‐Lite. Socially, the initial HRQoL gains following MBS are reinforced by improved social interactions, whereas weight regain may reactivate weight‐related stigma [51] and diminish social functioning. Together, these pathways demonstrate how weight regain undermines earlier HRQoL improvements and may explain the trajectory of partial deterioration after initial post‐surgical gains. Future longitudinal research should aim to disentangle these pathways, perhaps using mediation analyses to determine the relative contributions of physical symptom recurrence versus psychological distress to the overall decline in HRQoL during the weight regain phase.

The review and meta‐analysis by Nakanishi et al. was the first to determine the clinical relevance of HRQoL changes after BPD‐DS [18], demonstrating that post‐operative HRQoL achieved MCID, including PCS (but not MCS) of SF‐36, all SF‐36 subscales, IWQOL‐Lite score and LQ subscales [18]. The largest improvements were observed for the SF‐36 physical function and the LQ activity/mobility subscale [18]. However, high heterogeneity in outcomes limited interpretation. In addition to contributing to the findings established in the 2017 review [3], these results provide insights into the clinical relevance of BPD‐DS and the role of MBS in the management of obesity.

Evidence on post‐MBS HRQoL compared with community norms is conflicting. Raaijmakers et al. found post‐operative SF‐36 scores often exceeding norms, while pooled GIQLI remained below [24]. By contrast, Andersen et al. [52] in the 2017 SLR [3] reported post‐operative HRQoL, assessed with measures including the SF‐36 and QOLI, to be below norms after 5 years. Such discrepancies likely reflect heterogeneity in study design and patient‐reported outcomes (PROs).

Spilker's Quality of Life (QoL) pyramid helps explain this with obesity‐specific tools (e.g., IWQOL‐Lite) assessing function at the base, generic measures (e.g., SF‐36) capturing health status in the middle, and overall QoL instruments targeting life satisfaction at the apex [53]. The complexity of assessment increases higher up in the pyramid, making conflicting results more likely. This supports the use of both a well‐justified, validated generic instrument and an obesity‐specific tool to capture patient‐centred improvement and context at the population level.

Although there is strong evidence of short‐ and long‐term benefits of MBS on HRQoL, data with long‐term follow‐up (≥ 10 years) is very sparse. There is a critical need for extended follow‐ups in the field of MBS to capture the balance between long‐term benefits and risk complications, ensure data reliability and ultimately guide clinical decision‐making [54]. It is acknowledged that the implementation of extended follow‐up care presents significant challenges (e.g., dropouts, limited healthcare resources and infrastructure); however, addressing these barriers will help optimise patient care and advance MBS practices [54].

In addition to MBS, primary EBT has advanced significantly with the emergence of new endoscopic devices and refined techniques [48]. Emerging evidence suggests that EBT is effective in achieving clinically meaningful weight loss (≥ 10%) and improving metabolic syndrome in the short term, with reduced risks of complications and mortality due to its minimally invasive nature and reversibility compared with MBS [55]. EBT is recommended for use alongside dietary, exercise and behavioural interventions to achieve sustainable weight loss, especially when reversed [49]. The meta‐analysis by Gadd et al. showed significant HRQoL improvements at 6–76 months after IGB [22], but findings should be interpreted cautiously given the small number of studies, short to moderate follow‐ups, unadjusted confounders and low to very low quality of evidence across studies.

The meta‐analysis [23] that included studies with mixed designs (RCTs and non‐RCTs) focusing on exercise interventions detected no statistically significant effects on physical or mental health assessed by SF‐36 or SF‐12 in adults with obesity, likely due to short follow‐ups (< 16 weeks), diverse interventions (e.g., exercise type, intensity, duration), limited sample size and low quality of studies. In the current SLR, only one review limited to RCTs reported an association between weight loss through aerobic exercises and improved PCS and MCS of SF‐36 [17]. However, conclusions were hampered by the low credibility of evidence from one RCT, with poor generalisability of results to wider populations with obesity due to the trial [46] conducted in adults aged ≥ 65 years from a single centre. Of note, the current SLR identified another review of eight RCTs examining the impact of exercise added to energy restriction on HRQoL in adults with obesity who intended to lose weight [15]. However, the review [15] was excluded due to its BMI threshold for inclusion of ≥ 27.0 kg/m^2^ (Table S5). Although all eight RCTs included in the review were of excellent or good methodological quality, their evidence remained inconclusive regarding the additive effect of exercise during energy restriction on HRQoL despite weight loss benefits [15]. Notably, insights from the findings suggest the importance of using obesity‐specific instruments due to their higher sensitivity to changes in HRQoL compared with generic measures; that is, effect sizes demonstrated a small‐to‐large positive effect of exercise on IWQOL‐Lite total and domain scores, in contrast to inconsistent effect sizes of SF‐36 score and subscales across studies [15]. Collectively, these findings highlight the need for more high‐quality RCTs with long‐term follow‐ups in this research area. Nevertheless, lifestyle modifications with nutritional therapy and physical activity, as well as psychological and behavioural interventions, are recommended across clinical guidelines and remain the sustainable strategy for obesity management [56].

The non‐surgical treatment landscape for weight management is rapidly evolving, with emerging AOMs available for long‐term weight management [5]. A meta‐analysis of four RCTs that evaluated the efficacy and safety of once‐weekly semaglutide during 20–68 weeks of follow‐up in adults with overweight or obesity was identified from the search, with HRQoL pre‐specified as a secondary outcome [16]. However, the meta‐analysis was excluded as it included populations with BMI ≥ 27.0 kg/m^2^ (Table S5). Statistically significant HRQoL improvements (including SF‐36 physical functioning, PCS and MCS) were observed with once‐weekly semaglutide versus placebo in adults with overweight or obesity [16]. With the availability of AOMs, clinically meaningful weight loss becomes achievable, subsequently improving quality of life. The results warrant further SLRs focusing on the impact of AOMs on HRQoL in populations with obesity (BMI ≥ 30.0 kg/m^2^).

Overall, SF‐36 remains the most widely used generic measure (Table S8). Being developed and validated in the Medical Outcomes Study, SF‐36 allows for assessment of HRQoL associated with various medical conditions and for comparing HRQoL in patients with different diseases or with the general population (Table S2). Among obesity‐specific measures of HRQoL, IWQOL‐Lite was most frequently used (5/8 reviews; Table S8) [18, 21, 22, 23, 24]. The IWQOL‐Lite was developed based on heterogeneous populations who underwent various treatments for obesity, and was validated using hypothesis‐driven confirmatory factor analyses [29]. Reviews reporting IWQOL‐Lite consistently demonstrated a negative correlation between obesity and HRQoL, supporting the utility of IWQOL‐Lite in various populations with obesity. Nonetheless, using both generic and obesity‐specific measures is recommended to comprehensively capture different aspects of HRQoL affected by obesity.

Seven of eight included reviews exhibited ‘low’ to ‘critically low’ methodological quality and were downgraded primarily due to a lack of prospectively registered protocols and justification of excluded studies. Only one of four reviews with meta‐analyses was graded as ‘highly suggestive’, whereas two were rated down due to small sample size (*n* < 1000), high heterogeneity across studies or non‐significant results (*p* > 0.05).

RoB assessment was performed in all eight reviews, but quality/credibility/certainty of evidence was assessed in only two reviews [17, 22], with most studies rated as ‘low’ to ‘very low’ quality. As reported by Elsman et al. [57], SLRs on outcome measurement instruments often lack prospective protocol registrations, comprehensive search strategies and/or certainty assessments. Elsman et al. noted that conducting an SLR of outcome measurement instruments is challenging and requires standardised guidelines, like the recently updated COSMIN framework for SLRs [58], to facilitate high‐quality SLRs.

In summary, the findings from this updated SLR showed that obesity was strongly associated with impaired HRQoL, and individuals with an adverse metabolic profile may experience poorer HRQoL than metabolically healthy individuals. Significant HRQoL improvements associated with excess weight or BMI reductions following MBS were consistently demonstrated. Still, included studies provided insufficient evidence to suggest HRQoL improvements following non‐surgical weight‐loss interventions. Building on the evidence in the 2017 review, the findings from this review continue to show that there is a lack of standardisation in study design and reporting of PRO data in this area of research, with a limited number of studies employing appropriate PRO instruments (i.e., use of both generic and obesity‐specific measures) and poor methodological quality.

### 4.2 Strengths and Limitations

A key strength of this review is its prospectively registered protocol on PROSPERO. No protocol deviations occurred during the review process, thus minimising the RoB. This review was conducted in compliance with the PRISMA guidelines and the Cochrane Handbook for Systematic Reviews of Interventions. Methodological quality and credibility of evidence of included review articles were assessed to minimise the RoB when interpreting the results. An extensive search of five databases was carried out, and screening and data extraction were conducted by two reviewers to further reduce RoB. However, it is still possible that relevant studies may have been missed.

Other limitations include a small sample size (*n* = 8) and low quality of included reviews, which may hinder interpretation of results. There is also the possibility of publication bias due to the exclusion of non‐English articles. The narrative synthesis relied on reported results in the included reviews; thus, there was limited opportunity to query the data extracted from the studies.

The use of 36 different HRQoL instruments across eight reviews illustrates the considerable variation in measurement approaches within this field. Such heterogeneity makes evidence synthesis more challenging. Although standardisation could enhance comparability and strengthen the cumulative knowledge base, reaching agreement on a common set of instruments is itself a complex and contentious issue that has led to differing opinions [59, 60]. Ultimately, the methodological quality and contextual appropriateness of instruments may remain more important than uniformity per se.

### 4.3 Implications and Recommendations for Future Studies

Although this SLR covers the latest data published over 8 years since the original SLR of reviews [3], major advancements remain limited and significant knowledge gaps persist regarding the impact of obesity and weight management on HRQoL in adults, particularly concerning interactions between psychological, social and biological factors. Addressing these gaps requires innovative research, with new studies and updated SLRs on emerging interventions. Notably, no SLRs focusing on AOMs and HRQoL were identified despite the growing role of these treatments in obesity care, limiting conclusions on this emerging treatment area. Less restrictive PICOS criteria may be considered for future SLRs to allow for inclusion of reviews exploring AOMs that are indicated for adults with a BMI ≥ 27.0 kg/m^2^.

Many recommendations made in the original review remain unaddressed, such as reporting HRQoL outcomes with reference to clinical meaningfulness [3]. Although one included review [18] implemented this recommendation, future SLRs should ensure that outcome pre‐specification is sufficiently detailed and that results are presented in a clinically interpretable manner. To increase sensitivity and specificity, HRQoL assessments should combine validated generic and obesity‐specific instruments, enabling both norm‐based comparisons and the detection of patient‐centred changes [24]. Using disease‐specific PROs improves accuracy, informs interventions, guides policy and enhances patient outcomes [61]. To address the profound impact of measurement heterogeneity, future clinical trials and observational studies should adopt a dual‐instrument approach, employing at least one validated generic measure and one validated obesity‐specific measure. Furthermore, future SLRs in this domain should adhere to the COSMIN framework to ensure a rigorous and standardised assessment of the quality of the measurement instruments used in primary studies. A summary of additional recommendations for future studies and reviews is provided in Table 4.

**TABLE 4:** Summary of recommendations for future studies and review articles.

Category	List of recommendations
Review articles	A prospectively registered study protocol should be considered for SLRs and meta‐analyses to minimise RoB SLRs and meta‐analyses should be conducted in line with the existing guidelines (e.g., PRISMA guidelines and Cochrane Handbook for Systematic Reviews of Interventions) to ensure methodological quality and comprehensive reporting of results (including a list of excluded articles with reasons for exclusion) Alongside RoB and publication bias, assessment of quality of evidence should be performed to ensure conclusions are not biased For SLRs of reviews, reporting the degree of overlapping is highly recommended Primary outcomes and research questions should be pre‐specified in sufficient detail HRQoL analyses may formally consider the mediating effects of weight loss to understand whether the driver of HRQoL is weight loss itself or the weight‐loss intervention, where possible Interpretation of changes in HRQoL outcomes with reference to minimal clinically important difference may be considered to ensure the results are of clinical relevance Estimating effect size of changes in HRQoL allows for meaningful comparisons of results between studies and interpretation of results Reviews evaluating the effect of anti‐obesity medications on HRQoL are warranted
Research articles	Future studies should consider using at least one generic and one obesity‐specific measure of HRQoL Standardising the reporting of HRQoL by presenting all available scale scores should be considered to enhance the quality of research in this area. Ultimately, this will be beneficial for future SLRs and meta‐analyses by allowing for in‐depth, direct comparisons across studies

Abbreviations: HRQoL, health‐related quality of life; PRISMA, Preferred Reporting Items for Systematic reviews and Meta‐Analyses; RoB, risk of bias; SLR, systematic literature review.

Methodological quality for SLRs of PRO research must improve, particularly in defining research questions, registering protocols prospectively, applying robust search strategies and selection criteria, and using transparent evidence grading and reporting standards. Adoption of updated methodological frameworks, including COSMIN, will help address these gaps and strengthen the evidence base for HRQoL in obesity care [58, 62].

## Author Contributions

All authors contributed to the study design, and were involved in reviewing the literature, collating, analysing and interpreting the data, and drafting and critically revising the manuscript. All authors take full responsibility for the content of this manuscript.

## Ethics Statement

The authors have nothing to report.

## Conflicts of Interest

Tone Nygaard Flølo works as an Associate Professor at Oslo Metropolitan University, Oslo, Norway, and holds a position as a researcher at Haukeland University Hospital, Voss, Norway. Hui‐Hsuan Liu works as a Scientific Consultant at OPEN Health Communications, London, UK. John Roger Andersen works as a Professor at Western Norway University of Applied Sciences and holds a position as a researcher at Førde Hospital Trust, both in Førde, Norway. He has received honoraria from Novo Nordisk A/S for delivering lectures and participating in meetings on diabetes and obesity, which were organised and hosted by Novo Nordisk A/S for health professionals. Ronette L. Kolotkin works as a Consulting Professor at Duke University School of Medicine, Durham, NC, USA, and is a Professor Emerita at Western Norway University of Applied Sciences and at Vestfold Hospital Trust, Tønsberg, Norway. She is the Owner/Manager of Quality of Life Consulting, PLLC, Durham, NC, USA; and receives royalties from Duke University as an IWQOL‐Lite and IWQOL‐Lite‐CT developer, as well as support as a consultant to Novo Nordisk, Eli Lilly and Pfizer. She is also a shareholder of Novo Nordisk. The authors have no other relevant affiliations or financial involvement with any organisation or entity with a financial interest in or financial conflict with the subject matter or materials discussed in the manuscript apart from those disclosed.

## Supporting information


**Data S1:** cob70049‐sup‐0001‐supinfo1.docx.

**Data S2:** cob70049‐sup‐0002‐supinfo2.pdf.

## Data Availability

Data sharing not applicable to this article as no datasets were generated or analysed during the current study.

## References

[1] World Obesity Federation , “World Obesity Atlas 2024,” accessed January 1, 2025, https://www.worldobesity.org/news/world‐obesity‐atlas‐2024.

[2] K. R. Fontaine and I. Barofsky , “Obesity and Health‐Related Quality of Life,” Obesity Reviews 2, no. 3 (2001): 173–182, 10.1046/j.1467-789x.2001.00032.x. 12120102

[3] R. L. Kolotkin and J. R. Andersen , “A Systematic Review of Reviews: Exploring the Relationship Between Obesity, Weight Loss and Health‐Related Quality of Life,” Clinical Obesity 7, no. 5 (2017): 273–289, 10.1111/cob.12203. 28695722 PMC5600094

[4] J. Fastenau , R. L. Kolotkin , K. Fujioka , M. Alba , W. Canovatchel , and S. Traina , “A Call to Action to Inform Patient‐Centred Approaches to Obesity Management: Development of a Disease‐Illness Model,” Clinical Obesity 9, no. 3 (2019): e12309, 10.1111/cob.12309. 30977293 PMC6594134

[5] M. Chakhtoura , R. Haber , M. Ghezzawi , C. Rhayem , R. Tcheroyan , and C. S. Mantzoros , “Pharmacotherapy of Obesity: An Update on the Available Medications and Drugs Under Investigation,” EClinicalMedicine 58 (2023): 101882, 10.1016/j.eclinm.2023.101882. 36992862 PMC10041469

[6] D. Eisenberg , S. A. Shikora , E. Aarts , et al., “2022 American Society of Metabolic and Bariatric Surgery (ASMBS) and International Federation for the Surgery of Obesity and Metabolic Disorders (IFSO): Indications for Metabolic and Bariatric Surgery,” Obesity Surgery 33, no. 1 (2023): 3–14, 10.1007/s11695-022-06332-1. 36336720 PMC9834364

[7] A. M. Velardi , P. Anoldo , S. Nigro , and G. Navarra , “Advancements in Bariatric Surgery: A Comparative Review of Laparoscopic and Robotic Techniques,” Journal of Personalized Medicine 14, no. 2 (2024): 151, 10.3390/jpm14020151. 38392584 PMC10890254

[8] L. Irvin , L. A. Madden , P. Marshall , and R. V. Vince , “Digital Health Solutions for Weight Loss and Obesity: A Narrative Review,” Nutrients 15, no. 8 (2023): 1858, 10.3390/nu15081858. 37111077 PMC10145832

[9] National Institute for Health and Care Excellence , “Obesity: Identification, Assessment and Management. Clinical Guideline [CG189],” July 26, 2023, accessed April 15, 2024, https://www.nice.org.uk/guidance/cg189/chapter/Recommendations. 36719951

[10] E. Grunvald , R. Shah , R. Hernaez , et al., “AGA Clinical Practice Guideline on Pharmacological Interventions for Adults With Obesity,” Gastroenterology 163, no. 5 (2022): 1198–1225, 10.1053/j.gastro.2022.08.045. 36273831

[11] M. J. Page , J. E. McKenzie , P. M. Bossuyt , et al., “The PRISMA 2020 Statement: An Updated Guideline for Reporting Systematic Reviews,” BMJ (Clinical Research Ed.) 372 (2021): n71, 10.1136/bmj.n71. PMC8005924 33782057

[12] Cochrane , “Cochrane Handbook for Systematic Reviews of Interventions,” accessed April 17, 2024, https://training.cochrane.org/handbook/current.

[13] R. A. Speckman and J. L. Friedly , “Asking Structured, Answerable Clinical Questions Using the Population, Intervention/Comparator, Outcome (PICO) Framework,” PM & R: The Journal of Injury, Function, and Rehabilitation 11, no. 5 (2019): 548–553, 10.1002/pmrj.12116. 30729707

[14] National Institute for Health and Care Research , “The International Prospective Register of Systematic Reviews (PROSPERO),” accessed April 18, 2024, https://www.crd.york.ac.uk/prospero/.

[15] D. J. van den Hoek , C. T. Miller , S. F. Fraser , S. E. Selig , and J. B. Dixon , “Does Exercise Training Augment Improvements in Quality of Life Induced by Energy Restriction for Obese Populations? A Systematic Review,” Quality of Life Research 26, no. 10 (2017): 2593–2605, 10.1007/s11136-017-1602-9. 28551836

[16] P. Zhong , H. Zeng , M. Huang , W. Fu , and Z. Chen , “Efficacy and Safety of Once‐Weekly Semaglutide in Adults With Overweight or Obesity: A Meta‐Analysis,” Endocrine 75, no. 3 (2022): 718–724, 10.1007/s12020-021-02945-1. 34981419

[17] A. Jayedi , S. Soltani , A. Emadi , M.‐S. Zargar , and A. Najafi , “Aerobic Exercise and Weight Loss in Adults: A Systematic Review and Dose–Response Meta‐Analysis,” JAMA Network Open 7, no. 12 (2024): e2452185, 10.1001/jamanetworkopen.2024.52185. 39724371 PMC11672165

[18] H. Nakanishi , A. F. Teixeira , R. H. Matar , et al., “Impact on Mid‐Term Health‐Related Quality of Life After Duodenal Switch: A Systematic Review and Meta‐Analysis,” Obesity Surgery 33, no. 3 (2023): 769–779, 10.1007/s11695-022-06449-3. 36609744

[19] N. Vega‐Albornoz , O. Navarro‐Mora , and M. Á. López‐Espinoza , “Effect of Bariatric Surgery on Quality of Life in Obese Patients: A Global Systematic Review,” Revista de la Facultad de Medicina Humana 23, no. 4 (2023): 108–116, 10.25176/RFMH.v23i4.5727.

[20] B. Abiri , F. Hosseinpanah , S. Banihashem , S. A. Madinehzad , and M. Valizadeh , “Mental Health and Quality of Life in Different Obesity Phenotypes: A Systematic Review,” Health and Quality of Life Outcomes 20, no. 1 (2022): 63, 10.1186/s12955-022-01974-2. 35439997 PMC9019986

[21] R. Sierżantowicz , J. R. Ładny , and J. Lewko , “Quality of Life After Bariatric Surgery—A Systematic Review,” International Journal of Environmental Research and Public Health 19, no. 15 (2022): 9078, 10.3390/ijerph19159078. 35897447 PMC9330722

[22] N. Gadd , A. McIntosh , B. Fear‐Keen , J. Hoult , I. R. Maimone , and S. Marshall , “Do Endoscopic Bariatric Procedures Improve Postprocedural Quality of Life and Mental Health? A Systematic Review and Meta‐Analysis,” Obesity Surgery 30, no. 10 (2020): 4091–4100, 10.1007/s11695-020-04860-2. 32761319

[23] A. Baillot , S. Saunders , J. Brunet , A. J. Romain , A. Trottier , and P. Bernard , “A Systematic Review and Meta‐Analysis of the Effect of Exercise on Psychosocial Outcomes in Adults With Obesity: A Call for More Research,” Mental Health and Physical Activity 14 (2018): 1–10, 10.1016/j.mhpa.2017.12.004.

[24] L. C. Raaijmakers , S. Pouwels , S. E. Thomassen , and S. W. Nienhuijs , “Quality of Life and Bariatric Surgery: A Systematic Review of Short‐ and Long‐Term Results and Comparison With Community Norms,” European Journal of Clinical Nutrition 71, no. 4 (2017): 441–449, 10.1038/ejcn.2016.198. 27804961

[25] B. J. Shea , B. C. Reeves , G. Wells , et al., “AMSTAR 2: A Critical Appraisal Tool for Systematic Reviews That Include Randomised or Non‐Randomised Studies of Healthcare Interventions, or Both,” BMJ 358 (2017): j4008, 10.1136/bmj.j4008. 28935701 PMC5833365

[26] P. Fusar‐Poli and J. Radua , “Ten Simple Rules for Conducting Umbrella Reviews,” Evidence‐Based Mental Health 21, no. 3 (2018): 95–100, 10.1136/ebmental-2018-300014. 30006442 PMC10270421

[27] D. Pieper , S. L. Antoine , T. Mathes , E. A. Neugebauer , and M. Eikermann , “Systematic Review Finds Overlapping Reviews Were Not Mentioned in Every Other Overview,” Journal of Clinical Epidemiology 67, no. 4 (2014): 368–375, 10.1016/j.jclinepi.2013.11.007. 24581293

[28] R. L. Kolotkin and R. D. Crosby , “Psychometric Evaluation of the Impact of Weight on Quality of Life‐Lite Questionnaire (IWQOL‐Lite) in a Community Sample,” Quality of Life Research 11, no. 2 (2002): 157–171, 10.1023/a:1015081805439. 12018739

[29] R. L. Kolotkin , R. D. Crosby , K. D. Kosloski , and G. R. Williams , “Development of a Brief Measure to Assess Quality of Life in Obesity,” Obesity Research 9, no. 2 (2001): 102–111, 10.1038/oby.2001.13. 11316344

[30] R. L. Kolotkin , R. D. Crosby , and G. R. Williams , “Health‐Related Quality of Life Varies Among Obese Subgroups,” Obesity Research 10, no. 8 (2002): 748–756, 10.1038/oby.2002.102. 12181383

[31] R. L. Kolotkin , J. Kim , L. E. Davidson , R. D. Crosby , S. C. Hunt , and T. D. Adams , “12‐Year Trajectory of Health‐Related Quality of Life in Gastric Bypass Patients Versus Comparison Groups,” Surgery for Obesity and Related Diseases 14, no. 9 (2018): 1359–1365, 10.1016/j.soard.2018.04.019. 29884519

[32] G. W. Strain , M. H. Torghabeh , M. Gagner , et al., “The Impact of Biliopancreatic Diversion With Duodenal Switch (BPD/DS) Over 9 Years,” Obesity Surgery 27, no. 3 (2017): 787–794, 10.1007/s11695-016-2371-1. 27686233

[33] J. Karlsson , C. Taft , A. Rydén , L. Sjöström , and M. Sullivan , “Ten‐Year Trends in Health‐Related Quality of Life After Surgical and Conventional Treatment for Severe Obesity: The SOS Intervention Study,” International Journal of Obesity 31, no. 8 (2007): 1248–1261, 10.1038/sj.ijo.0803573. 17356530

[34] D. M. Felsenreich , G. Prager , R. Kefurt , et al., “Quality of Life 10 Years After Sleeve Gastrectomy: A Multicenter Study,” Obesity Facts 12, no. 2 (2019): 157–166, 10.1159/000496296. 30879011 PMC6547272

[35] A. Aasprang , J. R. Andersen , V. Våge , R. Kolotkin , and G. K. Natvig , “Ten‐Year Changes in Health‐Related Quality of Life After Biliopancreatic Diversion With Duodenal Switch,” Surgery for Obesity and Related Diseases 12, no. 8 (2016): 1594–1600, 10.1016/j.soard.2016.04.030. 27425783

[36] M. I. Duarte , D. P. Bassitt , O. C. Azevedo , J. Waisberg , N. Yamaguchi , and P. E. Pinto Junior , “Impact on Quality of Life, Weight Loss and Comorbidities: A Study Comparing the Biliopancreatic Diversion With Duodenal Switch and the Banded Roux‐en‐Y Gastric Bypass,” Arquivos de Gastroenterologia 51, no. 4 (2014): 320–327, 10.1590/s0004-28032014000400010. 25591161

[37] K. Elias , J. Hedberg , and M. Sundbom , “Prevalence and Impact of Acid‐Related Symptoms and Diarrhea in Patients Undergoing Roux‐en‐Y Gastric Bypass, Sleeve Gastrectomy, and Biliopancreatic Diversion With Duodenal Switch,” Surgery for Obesity and Related Diseases 16, no. 4 (2020): 520–527, 10.1016/j.soard.2019.12.020. 32057678

[38] G. W. Strain , R. L. Kolotkin , G. F. Dakin , et al., “The Effects of Weight Loss After Bariatric Surgery on Health‐Related Quality of Life and Depression,” Nutrition & Diabetes 4, no. 9 (2014): e132, 10.1038/nutd.2014.29. 25177912 PMC4183970

[39] P. Familiari , G. Costamagna , D. Bléro , et al., “Transoral Gastroplasty for Morbid Obesity: A Multicenter Trial With a 1‐Year Outcome,” Gastrointestinal Endoscopy 74, no. 6 (2011): 1248–1258, 10.1016/j.gie.2011.08.046. 22136774

[40] C. Moreno , J. Closset , S. Dugardeyn , et al., “Transoral Gastroplasty Is Safe, Feasible, and Induces Significant Weight Loss in Morbidly Obese Patients: Results of the Second Human Pilot Study,” Endoscopy 40, no. 5 (2008): 406–413, 10.1055/s-2007-995748. 18459077

[41] G. M. Hinnouho , A. Singh‐Manoux , A. Gueguen , et al., “Metabolically Healthy Obesity and Depressive Symptoms: 16‐Year Follow‐Up of the Gazel Cohort Study,” PLoS One 12, no. 4 (2017): e0174678, 10.1371/journal.pone.0174678. 28384219 PMC5383223

[42] H. Risstad , T. T. Søvik , M. Engström , et al., “Five‐Year Outcomes After Laparoscopic Gastric Bypass and Laparoscopic Duodenal Switch in Patients With Body Mass Index of 50 to 60: A Randomized Clinical Trial,” JAMA Surgery 150, no. 4 (2015): 352–361, 10.1001/jamasurg.2014.3579. 25650964

[43] T. I. Karlsen , R. S. Lund , J. Røislien , et al., “Health Related Quality of Life After Gastric Bypass or Intensive Lifestyle Intervention: A Controlled Clinical Study,” Health and Quality of Life Outcomes 11 (2013): 17, 10.1186/1477-7525-11-17. 23406190 PMC3599616

[44] L. M. Warkentin , S. R. Majumdar , J. A. Johnson , et al., “Weight Loss Required by the Severely Obese to Achieve Clinically Important Differences in Health‐Related Quality of Life: Two‐Year Prospective Cohort Study,” BMC Medicine 12 (2014): 175, 10.1186/s12916-014-0175-5. 25315502 PMC4212133

[45] H. E. Oria and M. K. Moorehead , “Updated Bariatric Analysis and Reporting Outcome System (BAROS),” Surgery for Obesity and Related Diseases 5, no. 1 (2009): 60–66, 10.1016/j.soard.2008.10.004. 19161935

[46] D. T. Villareal , L. Aguirre , A. B. Gurney , et al., “Aerobic or Resistance Exercise, or Both, in Dieting Obese Older Adults,” New England Journal of Medicine 376, no. 20 (2017): 1943–1955, 10.1056/NEJMoa1616338. 28514618 PMC5552187

[47] P. E. O'Brien , L. Brennan , C. Laurie , and W. Brown , “Intensive Medical Weight Loss or Laparoscopic Adjustable Gastric Banding in the Treatment of Mild to Moderate Obesity: Long‐Term Follow‐Up of a Prospective Randomised Trial,” Obesity Surgery 23, no. 9 (2013): 1345–1353, 10.1007/s11695-013-0990-3. 23760764

[48] J. I. Mechanick , C. Apovian , S. Brethauer , et al., “Clinical Practice Guidelines for the Perioperative Nutrition, Metabolic, and Nonsurgical Support of Patients Undergoing Bariatric Procedures—2019 Update: Cosponsored by American Association of Clinical Endocrinologists/American College of Endocrinology, The Obesity Society, American Society for Metabolic and Bariatric Surgery, Obesity Medicine Association, and American Society of Anesthesiologists,” Obesity 28, no. 4 (2020): O1–O58, 10.1002/oby.22719. 32202076

[49] B. K. Abu Dayyeh , S. Edmundowicz , and C. C. Thompson , “Clinical Practice Update: Expert Review on Endoscopic Bariatric Therapies,” Gastroenterology 152, no. 4 (2017): 716–729, 10.1053/j.gastro.2017.01.035. 28147221

[50] W. C. King , A. S. Hinerman , S. H. Belle , A. S. Wahed , and A. P. Courcoulas , “Comparison of the Performance of Common Measures of Weight Regain After Bariatric Surgery for Association With Clinical Outcomes,” Journal of the American Medical Association 320, no. 15 (2018): 1560–1569, 10.1001/jama.2018.14433. 30326125 PMC6233795

[51] L. Tolvanen , A. Christenson , P. J. Surkan , and Y. T. Lagerros , “Patients' Experiences of Weight Regain After Bariatric Surgery,” Obesity Surgery 32, no. 5 (2022): 1498–1507, 10.1007/s11695-022-05908-1. 35061154 PMC8986695

[52] J. R. Andersen , A. Aasprang , T. I. Karlsen , G. K. Natvig , V. Våge , and R. L. Kolotkin , “Health‐Related Quality of Life After Bariatric Surgery: A Systematic Review of Prospective Long‐Term Studies,” Surgery for Obesity and Related Diseases 11, no. 2 (2015): 466–473, 10.1016/j.soard.2014.10.027. 25820082

[53] B. Spilker , Quality of Life and Pharmacoeconomics in Clinical Trials (Lippincott‐Raven, 1996).

[54] A. Csendes and E. Cruz , “What Is Long‐Term Follow‐Up in Bariatric Surgery? A Proposal,” Obesity Surgery 35, no. 3 (2025): 671–673, 10.1007/s11695-025-07729-4. 39910019

[55] A. Mauro , F. Lusetti , D. Scalvini , et al., “A Comprehensive Review on Bariatric Endoscopy: Where We Are Now and Where We Are Going,” Medicina 59, no. 3 (2023): 636. 36984637 10.3390/medicina59030636 PMC10052707

[56] M. A. Cornier , “A Review of Current Guidelines for the Treatment of Obesity,” American Journal of Managed Care 28, no. 15 Suppl (2022): S288–S296, 10.37765/ajmc.2022.89292. 36525676

[57] E. B. M. Elsman , L. B. Mokkink , I. L. Abma , et al., “Methodological Quality of 100 Recent Systematic Reviews of Health‐Related Outcome Measurement Instruments: An Overview of Reviews,” Quality of Life Research 33, no. 10 (2024): 2593–2609, 10.1007/s11136-024-03706-z. 38961010 PMC11452433

[58] L. B. Mokkink , E. B. M. Elsman , and C. B. Terwee , “COSMIN Guideline for Systematic Reviews of Patient‐Reported Outcome Measures Version 2.0,” Quality of Life Research 33, no. 11 (2024): 2929–2939, 10.1007/s11136-024-03761-6. 39198348 PMC11541334

[59] P. J. Dijkhorst , C. E. E. de Vries , C. B. Terwee , et al., “A Core Set of Patient‐Reported Outcome Measures to Measure Quality of Life in Obesity Treatment Research,” Obesity Reviews 26, no. 2 (2025): e13849, 10.1111/obr.13849. 39419653

[60] M. E. Greene , R. E. Goldman , and M. M. Hutter , “Selection of Patient‐Reported Outcomes Measures for Implementation in the Metabolic and Bariatric Surgery Accreditation Quality Improvement Program,” Surgery for Obesity and Related Diseases 19, no. 8 (2023): 897–906, 10.1016/j.soard.2023.01.031. 37037688

[61] J. M. Bonsel , A. J. Itiola , A. S. Huberts , G. J. Bonsel , and H. Penton , “The Use of Patient‐Reported Outcome Measures to Improve Patient‐Related Outcomes—A Systematic Review,” Health and Quality of Life Outcomes 22, no. 1 (2024): 101, 10.1186/s12955-024-02312-4. 39593045 PMC11600902

[62] W. G. Hopkins and D. S. Rowlands , “Standardization and Other Approaches to Meta‐Analyze Differences in Means,” Statistics in Medicine 43, no. 16 (2024): 3092–3108, 10.1002/sim.10114. 38761102

