# Supplementary material for: Exploring the Relationship Between Obesity, Weight Loss and Health‐Related Quality of Life: An Updated Systematic Review of Reviews
Source: Clin Obes. 2025 Nov 3;16(1):e70049. doi: 10.1111/cob.70049 (PMC12705258; doi:10.1111/cob.70049)
Supplement: Supplementary file 2 — Data S2: cob70049‐sup‐0002‐supinfo2.pdf. [file COB-16-e70049-s002.pdf]

SUPPORTING INFORMATION

Exploring the relationship between obesity, weight loss, and health-related quality of life: An updated systematic review of reviews

Tone Nygaard Flølo[1,2], Hui-Hsuan Liu[3], John Roger Andersen[4,5], Ronette L. Kolotkin[6,7,8,9]

[1]Department of Nursing and Health Promotion, Oslo Metropolitan University, Oslo, Norway

[2]Department of Surgery, Voss Hospital, Haukeland University Hospital, Voss, Norway

[3]Real-World Evidence, OPEN Health Communications, London, UK

[4]Faculty of Health and Social Sciences, Western Norway University of Applied Sciences, Førde, Norway

[5]Center of Health Research, Førde Hospital Trust, Førde, Norway

[6]Duke Family Medicine and Community Health, Duke University School of Medicine, Durham, NC, USA

[7]Western Norway University of Applied Sciences, Førde, Norway

[8]Morbid Obesity Centre, Vestfold Hospital Trust, Tønsberg, Norway

[9]Quality of Life Consulting, PLLC, Durham, NC, USA

Correspondence

Tone Nygaard Flølo, Department of Nursing and Health Promotion, Oslo Metropolitan University, Pilestredet Park 0890, 0176 Oslo, Norway

Tel: +47 672 36 451

Email: tonenyga@oslomet.no

TABLE S1. PICOS criteria

PICOS	Inclusion criteria	Exclusion criteria
Population	Adults aged ≥18 years with obesity (BMI ≥30 kg/m^2)	• Population not listed in the inclusion criteria<br>• Exclusive review of pediatric populations<br>• Exclusive review of populations with a specific comorbidity of obesity<br>• Exclusive review of pregnant women
Intervention	Any interventions for the management of adult obesity recommended across different international clinical practice guidelines, including, but not limited to, UK (NICE[1]), US (e.g. AHA/ACC/TOS,[2] AACE/ACE,[3] AGA,[4] ASMBS,[5] Endocrine Society[6]), Canadian,[7,8] and European[9] guidelines<br>• Lifestyle interventions<br>• Psychological and behavioral interventions<br>• Physical activity<br>• Dietary interventions<br>• Pharmacotherapy<br>• Metabolic and bariatric surgery	Interventions not listed in the inclusion criteria
Comparison	Any of the above interventions of interest	Comparators not listed in the inclusion criteria
Outcomes	Impact of obesity and/or weight management on HRQoL evaluated by validated questionnaires (including but not limited to the list provided in **Table S2**)<br>• Generic measures<br>• Obesity-specific measures (e.g. OP, IWQOL-Lite, IWQOL-Lite Clinical Trials Version, OWQOL, WRSM, M-A QOLQII, BODY-Q, PROS)<br>• Combined generic/obesity-specific measures	• Outcomes not listed in the inclusion criteria<br>• Studies that do not answer the review question: "what is the impact of obesity and/or weight management on QoL?" or include a brief mention only of how QoL is affected<br>• Studies that exclusively review HRQoL outcomes of a specific obesity-related comorbidity
Study design	Systematic reviews and/or meta-analyses	• Not a systematic review or meta-analysis<br>• Studies included in the previous systematic review
Time frame	From January 1, 2001 to current date	
Countries	No restrictions	
Language	English	Non-English publications

Abbreviations: AACE/ACE, American Association of Clinical Endocrinology/American College of Endocrinology; AGA, American Gastroenterological Association; AHA/ACC/TOS, American College of Cardiology/American Heart Association/The Obesity Society; ASMBS, American Society for Metabolic and Bariatric Surgery; BMI, body mass index; HRQoL, health-related quality of life; IWQOL, Impact of

Weight on Quality of Life; NICE, National Institute for Health and Care Excellence; M-A QOLQII, Moorehead–Ardelt Quality of Life; OP, Obesity-Related Problems Scale; OWQOL, Obesity and Weight Loss Quality of Life; PICOS, Population, Intervention, Comparison, Outcomes and Study design; PROS, Patient-Reported Outcomes in Obesity; QoL, quality of life; WRSM, Weight-Related Symptom Measure.

TABLE S2. List of health-related quality of life measures

Assessment (reference [author, date])	Abbreviation	Country of origin	Number of Items and domains	Description
**Generic measures**				
Visual analog scale (Scott and Huskisson, 1976)[10]	VAS	Unknown	• Varies	• A horizontal line anchored by word descriptors at each end<br>• Attempts to measure a characteristic or attitude that ranges across a continuum of values
General Well-Being Schedule (Fazio, 1977)[11]	GWB	USA	• 3 versions (18, 22, and 33 items)<br>○ 6 domains<br>▪ Anxiety<br>▪ Depression<br>▪ Positive well-being<br>▪ Self-control<br>▪ Vitality<br>▪ General health	• A brief questionnaire measuring an individual's subjective sense of well-being and distress over the preceding month
General Health Questionnaire (Goldberg, 1978)[12]	GHQ	England	• 60 items	• Developed as a screening measure to identify psychological distress among adults in primary care settings
General Health Ratings Index (Davies and Ware, 1981)[13]	GHRI	USA	• 26 items<br>○ 6 dimensions<br>▪ Current health<br>▪ Prior health<br>▪ Health outlook<br>▪ Resistance to illness<br>▪ Health worry/concern<br>▪ Sickness orientation	• A shorter version of the Health Perception Questionnaire

Nottingham Health Profile (Hunt et al., 1981)[14]	NHP	England	Part 1:<br>• 38 items<br>o 6 domains<br>▪ Energy level<br>▪ Pain<br>▪ Emotional reaction<br>▪ Sleep<br>▪ Social isolation<br>▪ Physical abilities<br>Part 2:<br>• 7 items	• Intended for primary healthcare providers to provide a brief indication of a patient's perceived emotional, social, and physical health problems
Sickness Impact Profile (Bergner et al., 1981)[15]	SIP	USA	• 136 items<br>• 12 categories<br>o Sleep and rest<br>o Emotional behavior<br>o Body care and movement<br>o Home management<br>o Mobility<br>o Social interaction<br>o Ambulation<br>o Alertness behavior<br>o Communication<br>o Work<br>o Recreation and pastimes<br>o Eating	• A 136-item self- or interviewer-administered, behaviorally based, health status questionnaire
Dartmouth Primary Care Cooperative Research Network and the World Organization of National Colleges, Academies, and Academic Associations of General Practitioners/Family	COOP-WONCA charts	USA	• 6 core charts of functional status<br>o Physical fitness<br>o Feelings<br>o Daily activities<br>o Social activities<br>o Change in health<br>o Overall health<br>o Pain (optional aspect)<br>o Sleep (optional aspect)	• A 6 single-item questionnaire to determine functional ability in chronically ill patients<br>• The average time for completion is <5 minutes<br>• A self-administered or interview-based questionnaire, during or after face-to-face contacts; scored by patients themselves, proxies, nurses, doctors, or other healthcare providers

Physicians (Nelson et al., 1990; van Weel, 1995)[16,17]				• Available in multiple languages
RAND 36-Item Health Survey (Version 1.0) (Hays et al., 1993; Hays and Morales, 2001)[18,19]	RAND-36	USA	• 36 items<br>• 8 domains<br>○ Physical functioning<br>○ Bodily pain<br>○ Role limitations due to physical health problems<br>○ Role limitations due to personal or emotional problems<br>○ Emotional well-being<br>○ Social functioning<br>○ Energy/fatigue<br>○ General health perceptions	• An identical questionnaire to SF-36, with a simpler and more straightforward scoring method/procedure[20]<br>• A profile measure with 36 questions that yields an 8-scale score and 2 summary scores (physical and mental health)
12-Item Short Form Survey (Ware et al., 1996)[21]	SF-12	USA	• 12 items<br>○ Physical component summary<br>○ Mental component summary	• A 1–2-page, 2-minute survey that yields summary physical and mental scores that are interchangeable with those from the SF-36
Medical Outcomes Study 36-Item Short Form Health Survey (Ware et al., 2000)[22]	SF-36	USA	• 36 items<br>• 8 domains<br>○ 4 physical HRQoL<br>▪ Physical functioning<br>▪ Physical role functioning<br>▪ Bodily pain<br>▪ General health<br>○ 4 mental HRQoL<br>▪ Vitality<br>▪ Social functioning<br>▪ Emotional role functioning<br>▪ Mental health	• A comprehensive short form with 36 questions that yields an 8-scale health profile as well as summary measures of HRQoL (physical component summary and mental component summary)
15D (Sintonen, 2001)[23]	15D	Australia	• 15 dimensions (and 15 items)<br>○ Breathing<br>○ Mental function<br>○ Speech (communication)<br>○ Vision	• The 15D is a generic, 15-dimensional measure of HRQoL (physical, social, and mental domains) that can be used as a single index score or as a profile of these dimensions

			○ Mobility<br>○ Usual activities<br>○ Vitality<br>○ Hearing<br>○ Eating<br>○ Elimination<br>○ Sleeping<br>○ Distress<br>○ Discomfort and symptoms<br>○ Sexual activity<br>○ Depression<br>• Each dimension is divided into 5 levels	
Quality of Well-Being Scale (Seiber et al., 2008)[24]	QWB	USA	• 71 items<br>• 4 domains<br>○ Physical activities<br>○ Social activities<br>○ Mobility<br>○ Symptom/problem complexes	• An interviewer-administered measure that measures well-being in individuals based on a person's level of functioning
EuroQoL-5 Dimension (Gusi et al., 2010)[25]	EQ-5D	UK, Finland, the Netherlands, Norway, and Sweden	• 5 domains<br>○ Mobility<br>○ Self-care<br>○ Usual activities<br>○ Pain/discomfort<br>○ Anxiety/depression	• A cognitively simple questionnaire suited for use in postal surveys, clinics, and face-to-face interviews
World Health Organization Quality-of-Life Scale (World Health Organization, 2012)[26]	WHOQOL	Switzerland	WHOQOL-Brief Version (BREF)[27]<br>• 26 items<br>• 4 domains<br>○ Physical health<br>○ Psychological domain<br>○ Social relationships<br>○ Environment	• Developed using a unique cross-cultural approach<br>• Available in 30 languages<br>• To assess individuals' perceptions of their position in life in the context of the culture and value systems in which they live and in relation to their goals, expectations, standards, and concerns
**Obesity-specific measures**				

Obesity-Related Problems Scale (Sullivan et al., 1993)[28]	OP	Sweden	• 8 items<br>○ Psychosocial problems related to weight	• Developed as part of the SOS large-scale prospective, matched (non-randomized) intervention trial of obesity examining psychosocial functioning and health before and after bariatric surgery<br>• The OP scale, a subset of the SOS measure described below, assesses the impact of obesity on psychosocial functioning<br>• Patients are asked how bothered they are by their obesity in specific situations<br>• Available in multiple languages
Impact of Weight on Quality of Life (Kolotkin et al., 1995)[29]	IWQOL	USA	• 74 items<br>○ 8 domains<br>▪ Health<br>▪ Social/interpersonal life<br>▪ Work<br>▪ Mobility<br>▪ Self-esteem<br>▪ Sexual life<br>▪ Activities of daily living<br>▪ Comfort with food	• Developed by obesity practitioners with input from patients attending an intensive lifestyle program<br>• Large number of items creates heavy respondent burden<br>• Test–retest reliability of only 1 day<br>• Not recommended—use IWQOL-Lite instead
Obesity-Specific Quality of Life (Le Pen et al., 1998)[30]	OSQOL	France	• 11 items<br>○ 4 domains<br>▪ Physical state<br>▪ Vitality/desire to do things<br>▪ Relations with other people<br>▪ Psychological state	• Developed in a large community sample<br>• Differentiates between people with and without obesity on 3 domains<br>• Not widely used
Obesity Related Well-Being (Mannucci et al., 1999)[31]	ORWELL 97	Italy	• 18 items<br>○ 2 factors<br>▪ Psychological status/social adjustment<br>▪ Physical symptoms/impairment	• Assesses relevance of symptoms plus occurrence<br>• Not widely used
Obesity Adjustment Survey-Short Form (Butler et al., 1999)[32]	OAS-SF	Canada	• 20 items<br>○ Overall adjustment to morbid obesity/psychological distress	• Detects change after bariatric surgery<br>• Limited to measuring psychological distress<br>• Development sample was predominantly female patients

				• Not widely used
Impact of Weight on Quality of Life-Lite (Kolotkin et al., 2001)[33]	IWQOL-Lite	USA	• 31 items<br>○ 5 domains<br>▪ Physical function<br>▪ Self-esteem<br>▪ Sexual life<br>▪ Public distress<br>▪ Work<br>○ Total score	• Demonstrates, reliability, validity, and responsiveness in numerous studies<br>• Scale structure verified with confirmatory factor analysis<br>• Scoring was originally based on raw scores but changed to a 0–100 scale in 2002 to facilitate interpretation of scores[34]<br>• Available in multiple languages
Obesity and Weight Loss Quality of Life (Niero et al., 2002)[35]	OWLQOL	USA and Europe	• 17 items producing a single score measuring a unitary concept of QoL related to obesity or weight loss	• Developed on a very large sample of people with obesity in the USA and Europe, with the aim of being multicultural<br>• Good internal consistency, test–retest reliability, validity, and responsiveness<br>• Intended to be used in conjunction with WRSM<br>• Available in multiple languages
Weight-Related Symptom Measure (Niero et al., 2002)[35]	WRSM	USA and Europe	• 20 items assessing symptoms associated with obesity or weight loss	• Developed on a very large sample of people with obesity in the USA and Europe, with the aim of being multicultural<br>• Good internal consistency, test–retest reliability, validity, and responsiveness<br>• Intended to be used in conjunction with OWLQOL<br>• Available in multiple languages
Moorehead–Ardelt Quality of Life Questionnaire II (Moorehead et al., 2003)[36]	M-A QoLQII	USA and Austria	• 6 items<br>○ General self-esteem<br>○ Physical activity<br>○ Social contacts<br>○ Satisfaction concerning work<br>○ Pleasure relating to sexuality<br>○ Eating behavior	• Update of the original M-A QoLQII<br>• Designed as a standalone measure and/or to be used in conjunction with BAROS<br>• Response options are anchored with graphics (e.g. smiley face)<br>• Available in multiple languages

Quality of Life, Obesity and Dietetics Questionnaire (Ziegler et al., 2005)[37]	QOLOD	France	• 36 items<br>• 5 domains<br>○ Physical impact<br>○ Psychosocial impact<br>○ Sex life<br>○ Comfort with food<br>○ Diet experience	• Developed and validated as a specific questionnaire usable in clinical trials in France<br>• Not widely used
Laval Questionnaire (Therrien et al., 2011)[38]	LQ	Canada	• 44 items<br>• 6 domains<br>○ Symptoms<br>○ Activity/mobility<br>○ Personal hygiene/clothing<br>○ Emotions<br>○ Social interactions<br>○ Sexual life	• Developed at a bariatric surgery center on 67 surgery patients and 45 wait-list patients<br>• Designed for use in clinical trials<br>• Developed in French; also available in Italian and English<br>• Reported responsiveness to change, validity, and reliability in one study<br>• Not widely used
Bariatric and Obesity-Specific Survey (Tayyem et al., 2014)[39]	BOSS	Scotland	• 42 items<br>• 6 domains<br>○ Incapacity<br>○ Work and well-being<br>○ Social function<br>○ Appearance and health<br>○ Eating patterns<br>○ Sexual health	• Based on data from a single center of 83 patients considering bariatric surgery, 68 who had undergone bariatric surgery, and 85 healthy volunteers<br>• One study reported good internal consistency and test–retest reliability and validity<br>• Not widely used
BODY-Q scales (de Vries et al., 2021; Klassen et al., 2016)[40,41]	BODY-Q	Canada, UK, and USA	• 29 independently functioning scales related to appearance, QoL, eating concerns, and quality of care<br>○ Body, abdomen, arms, back, buttocks, hips and outer thighs, inner thighs, skin, scars, body image, physical, psychological, sexual, social, symptoms, doctor, information, medical team, office staff<br>○ New scales were added in 2021 (de Vries et al.): expectations, eating behavior, eating-related distress, work life, eating-related symptoms	• Developed on a mixed sample of bariatric surgery patients, individuals interested in (or having had) surgical or nonsurgical body contouring<br>• Used Rasch Measurement methods to identify scales<br>• Available in several languages

IWQOL-Lite Clinical Trials Version (Kolotkin et al., 2017; 2019; 2021)[42-44]	IWQOL-Lite-CT	USA	• 20 items<br>• 2 domains<br>○ Physical (subset is physical function)<br>○ Psychosocial	• Items were designed for use in clinical trials for obesity<br>• Item development was based on input from clinical experts, qualitative research conducted with patients, as well as feedback from the U.S. Food and Drug Administration<br>• Demonstrated reliability, validity, and responsiveness in clinical trials<br>• Scale structure verified with confirmatory factor analysis<br>• Available in multiple languages
Patient-Reported Outcomes in Obesity (Aasprang et al., 2019)[45]	PROS	Norway	• 8 items<br>○ Common physical activities<br>○ Bodily pain<br>○ Discrimination or discourteous behavior<br>○ Sleep<br>○ Sexual life<br>○ Normal social interaction<br>○ Work, school, or other daily activities<br>○ Self-esteem	• PROS was designed to be easily administered and scored for use in clinical settings<br>• Demonstrated reliability, validity, and sensitivity to change over time<br>• Available in several languages
**Combined generic and obesity-specific measures**				
Swedish Obese Subjects (SOS) quality of life survey (Sullivan et al., 1993)[28]	SOS	Sweden	The SOS was a battery of measures that included the following:<br>• Current Health (CH) from the General Health Rating Index (GHRI)<br>• Short Form of the Mood Adjective Check list (MACL) to assess mental health<br>• Hospital Anxiety and Depression Scale (HADS)<br>• The Social interaction (SI) category from the Sickness Impact Profile (SIP)<br>• OP questionnaire (described above under obesity-specific measures)	• Developed as part of the SOS large-scale studies<br>• A battery of validated questionnaires used to yield general health and psychosocial functioning profile

Health-Related Quality of Life - Health State Preference (Mathias et al., 1997)[46]	HRQOL-HSP (or 'Lewin-TAG')	USA	• 55 items<br>○ 7 domains<br>▪ General health<br>▪ Comparative health<br>▪ Overweight distress<br>▪ Depression<br>▪ Self-regard<br>▪ Physical appearance<br>▪ Health state preference	HRQoL measure containing global and obesity-specific domains, and an obesity-specific HSP assessment, suitable for use with those who are classed as medically obese (BMI 30–43 kg/m^2)
**Combined obesity-specific and bariatric surgery-specific measures**				
Bariatric Quality of Life (Weiner et al., 2005; 2009)[47,48]	BQL	Germany	• 19 items<br>• 3 factors<br>○ General QoL<br>○ Comorbidities<br>○ GI side effects	• Developed on bariatric surgery patients, general surgery patients, and healthy volunteers<br>• A factor analysis in 2009 proposed a one-factor solution that accounted for 45.37% of the variance<br>• Has been described as deficient in content validity<br>• Available in German and English

Abbreviations: BAROS, Bariatric Analysis and Reporting System; BMI, body mass index; GI, gastrointestinal; HRQoL, health-related quality of life; QoL, quality of life.

TABLE S3. Search strategy for PubMed

Topic	#	Terms	Hits
Indication	#1	"obesity"[mesh] OR "obese"[all fields] OR "obesity"[all fields]	507,197
Outcomes of interest	#2	"quality of life"[mesh] OR "quality of life"[tiab] OR "health related quality of life"[tiab] OR "life quality"[tiab] OR "HRQoL"[tiab] OR "HQOL"[tiab] OR "QoL"[tiab]	510,259
String combination	#3	#1 AND #2	13,466
Filters	#4	#3 AND "systematic", "meta-analysis", "humans", "English language", "publication date from 01/01/2001 to present"	526

Search date: April 15, 2025.

TABLE S4. Search strategy for MEDLINE, Embase, and PsycInfo via ProQuest

Topic	Set	Terms	Hits
Indication	S1	All fields(“obesity” OR “obese”)	1,456,567
	S2	TI,AB(“quality of life” OR “health related quality of life” OR “HRQOL” OR “QoL” OR “HQOL” OR “life quality”)	1,203,518
	S3	S1 AND S2	38,193
Study design	S4	TI,AB(“systematic literature review” OR “meta-analysis” or “cochrane review”)	759,757
String combination	S5	S3 AND S4	576
Limits	S6	Limited by “humans”, “publication date after 01 January 2001”, and “English language”	540*

*Duplicates are removed from the search and from the result count.

Search date: April 15, 2025.

TABLE S5. Search strategy for CINAHL

Topic	Set	Terms	Hits
Indication	S1	All fields (“obesity” OR “obese”)	168,298
	S2	TI(“quality of life” OR “health related quality of life” OR “HRQOL” OR “QoL” OR “HQOL” OR “life quality”) OR AB(“quality of life” OR “health related quality of life” OR “HRQOL” OR “QoL” OR “HQOL” OR “life quality”)	170,726
	S3	S1 AND S2	4062
Study design	S4	TI(“systematic literature review” OR “meta-analysis” or “cochrane review”) OR AB(“systematic literature review” OR “meta-analysis” or “cochrane review”)	121,224
String combination	S5	S3 AND S4	85
Limits	S6	Limited by “humans”, “publication date after 01 January 2001” and “English language”	55

Search date: April 15, 2025

TABLE S6. Excluded articles with reasons for exclusion during full-text screening

Reference (author, year)	Reason for exclusion
Zhong et al., 2022[49]	Inclusion of population with overweight (BMI ≥27 kg/m^2)
van den Hoek et al., 2017[50]	Inclusion of population with overweight (BMI ≥27 kg/m^2) and obesity
Rajaie et al., 2022[51]	Inclusion of population with overweight (BMI ≥25 kg/m^2) and obesity
Lasikiewicz et al., 2014[52]	Inclusion of population with overweight (BMI ≥25 kg/m^2) and obesity (up to BMI of 45 kg/m^2)
Jones et al., 2021[53]	Inclusion of adults (aged ≥18 years) with overweight or obesity (BMI ≥25 kg/m^2) seeking intentional weight loss
Vosadi et al., 2025[54]	Inclusion of population with overweight (BMI ≥25 kg/m^2) and/or obesity
Hayes et al., 2017[55]	Evaluating whether weight changes (weight gain or loss) in adults and children with and without obesity is longitudinally associated with HRQoL outcomes, compared with those with a stable weight
Wu et al., 2020[56]	Comparison of the effect of different interventions or procedures on HRQoL
Małczak et al., 2021[57]	Comparison of HRQoL after different bariatric procedures
Lei et al., 2024[58]	Comparison of HRQoL after different bariatric procedures
Hu et al., 2020[59]	Comparison of HRQoL after different bariatric procedures
García-Honores et al., 2023[60]	Comparison of HRQoL after different bariatric procedures; did not report the effect of each intervention on HRQoL
Ganam et al., 2024	Assessing HRQoL and procedure outcomes in patients who have undergone the conversion from SG to RYGB
Buckell et al., 2021[62]	No SLR or meta-analysis was carried out; the study identified relevant RCTs from two key SLRs and used longitudinal data from five large RCTs of behavioral weight-loss interventions in an individual participant data meta-analysis
Jobanputra et al., 2023[63]	Focusing on the effect of licensed weight-loss pharmacotherapies on physical function and fitness
Stewart and Avenell, 2016[64]	Did not report HRQoL due to lack of data
Asiah et al., 2023[65]	Did not report HRQoL
Adil et al., 2018[66]	Did not report HRQoL
Colquitt et al., 2014[67]	Inclusion of population with overweight; did not report HRQoL
Colquitt et al., 2009[68]	Did not report HRQoL
Martín-Mariscal, 2018[69]	Published in Spanish and appears to be an abstract or short article
Romantsova, 2020[70]	Conference abstract
Vasconcelos et al., 2016[71]	Conference abstract

Abbreviations: BMI, body mass index; HRQoL, health-related quality of life; RCT, randomized controlled trial; RYGB, Roux-en-Y gastric bypass; SG, sleeve gastrectomy; SLR, systematic literature review.

TABLE S7. Overlap of studies included in the reviews

Reference (author, date)	Vega-Albornoz et al., 2023[72]	Sierżantowicz et al., 2022[73]	Nakanishi et al., 2023[74]	Gadd et al., 2020[75]	Abiri et al., 2022[76]	Baillot et al., 2018[77]	Raaijmakers et al., 2017[78]	Jayedi et al., 2024[79]
Herring et al., 2016[80]	x							
Raaijmakers et al., 2017[78]	x							
Sierżantowicz et al., 2022[73]	x							
Karlsson et al., 2007[81]		x					x	
Kinzl et al., 2011[82]		x						
O'Brien et al., 2013[83]		x						
Aarts et al., 2014[84]		x						
Canetti et al., 2016[85]		x						
Herpertz et al., 2015[86]		x						
Aasprang et al., 2016[87]		x	x					
Strain et al., 2017[88]		x						
Nguyen et al., 2018[89]		x						
Kolotkin et al., 2018[90]		x						
Rolim et al., 2018[91]		x						
Galli et al., 2018[92]		x						
Askari et al., 2020[93]		x						
Neovius et al., 2018[94]		x						
Bruze et al., 2018[95]		x						
Legenbauer et al., 2019[96]		x						
Mabey et al., 2021[97]		x						

Felsenreich et al., 2019[98]	x		
Badaoui et al., 2021[99]		x	
Biron et al., 2018[100]		x	
Duarte et al., 2014[101]		x	
Elias et al., 2020[102]		x	
Khandalavala et al., 2016[103]		x	
Laurenius et al., 2010[104]		x	
Magee et al., 2011[105]		x	
Malo et al., 2020[106]		x	
Strain et al., 2014[107]		x	
Uribarri-Gonzalez et al., 2023[108]		x	
Weiner et al., 2005[109]		x	
Ahmed and Ezzat, 2019[110]			x
De Castro et al., 2010[111]			x
Deliopoulou et al., 2013[112]			x
Familiari et al., 2011[113]			x
Fiorillo et al., 2020[114]			x
Fuller et al., 2013[115]			x
Alfredo et al., 2014[116]			x
Guedes et al., 2019[117]			x
Marinos et al., 2014[118]			x
Machytka et al., 2017[119]			x
Moreno et al., 2008[120]			x
Mui et al., 2010[121]			x

Ponce et al., 2013[122]	x	
Norén and Forssell, 2016[123]	x	
Tayyem et al., 2011[124]	x	
Tayyem et al., 2014[39]	x	
Guedes et al., 2016[125]	x	
Raftopoulos et al., 2017[126]	x	
Reimão et al., 2018[127]	x	
Thompson et al., 2017[128]	x	
Guedes et al., 2017[129]	x	
Mehrabi et al., 2021[130]		x
Kim et al., 2020[131]		x
Donini et al., 2016[132]		x
Lopez-Garcia et al., 2017[133]		x
Tsai et al., 2008[134]		x
Phillips and Perry, 2015[135]		x
Yang et al., 2016[136]		x
Truthmann et al., 2017[137]		x
Delgado et al., 2018[138]		x
Portugal-Nunes et al., 2021[139]		x
Seo et al., 2020[140]		x
Imbiriba et al., 2021[141]		x
Yosaee et al., 2018[142]		x
Hamer et al., 2012[143]		x
Park and Lee, 2021[144]		x

Hinnouho et al., 2017[145]	x	
Amiri et al., 2018[146]	x	
Ul-Haq et al., 2012[147]	x	
Abou-Raya et al., 2014[148]		x
Christensen et al., 2015[149]		x
Horwich et al., 2011[150]		x
Imayama et al., 2011[151]		x
Kitzman et al., 2016[152]		x
Labrunée et al., 2012[153]		x
Casilda-López et al., 2017[154]		x
Megakli et al., 2016[155]		x
Megakli et al., 2017[156]		x
Napoli et al., 2014[157]		x
Nieman et al., 2000[158]		x
Plotnikoff et al., 2010[159]		x
Sarsan et al., 2006[160]		x
Sukala et al., 2013[161]		x
Svensson et al., 2017[162]		x
Vasconcelos et al., 2016[71]		x
Villareal et al., 2011[163]		x
Rica et al., 2013[164]		x
Baillot et al., 2012[165]		x
del Rey-Moya et al., 2013[166]		x
Lucha-López et al., 2012[167]		x
Muller-Pinget et al., 2012[168]		x

Wouters et al., 2010[169]	x	
Grans et al., 2012[170]		x
Klingemann et al., 2009[171]		x
Lier et al., 2011[172]		x
Martínez et al., 2010[173]		x
Freys et al., 2001[174]		x
van Hout et al., 2009[175]		x
Aarts et al., 2014[176]		x
Aasprang et al., 2013[177]		x
Adami et al., 2005[178]		x
Adams et al., 2010[179]		x
Adams et al., 2012[180]		x
Ahroni et al., 2005[181]		x
Boan et al., 2004[182]		x
Brunault et al., 2015[183]		x
Brunault et al., 2011[184]		x
Busetto et al., 2015[185]		x
Charalampakis et al., 2015[186]		x
Efthymiou et al., 2015[187]		x
Fezzi et al., 2011[188]		x
Hansen et al., 2014[189]		x
Helmiö et al., 2011[190]		x
Julia et al., 2013[191]		x
Karlsen et al., 2013[192]		x

Kolotkin et al., 2009[193]							x	
Kolotkin et al., 2012[194]							x	
Lee et al., 2002[195]							x	
Major et al., 2015[196]							x	
Mar et al., 2013[197]							x	
Mathus-Vliegen and de Wit, 2007[198]							x	
Nadalini et al., 2014[199]							x	
O'Brien et al., 2005[200]							x	
Omotosho et al., 2016[201]							x	
Peterli et al., 2013[202]							x	
Pilone et al., 2012[203]							x	
Risstad et al., 2015[204]							x	
Suter et al., 2011[205]							x	
Warkentin et al., 2014[206]							x	
White et al., 2015[207]							x	
Zijlstra et al., 2013[208]							x	
Villareal et al., 2017[209]								x
**Number of included studies in each review (*k*)**	**3**	**18**	**12**	**21**	**18**	**23**	**40**	**1**

**Total number of included studies (including double counting, N)**	**Number of rows (r)**	**Number of reviews (c)**	**% overlap**	**CA =** $\frac{N}{r \times c}$	**CCA =** $\frac{N-r}{(r \times c)-r}$
136	134	8	1.49%	0.13	0.0021

Abbreviations: CA, covered area; CCA, corrected covered area.

TABLE S8. HRQoL measures used across studies included in each review

<table>
<thead>
<tr><th>HRQoL measure</th><th>Jayedi et al., 2024[79]</th><th>Nakanishi et al., 2023[74]</th><th>Vega-Albornoz et al., 2023[72]</th><th>Abiri et al., 2022[76]</th><th>Sierżantowicz et al., 2022[73]</th><th>Gadd et al., 2020[75]</th><th>Baillot et al., 2018[77]</th><th>Raaijmakers et al., 2017[78]</th></tr>
</thead>
<tbody>
<tr><td colspan="9">Generic measures (n = 8 types)</td></tr>
<tr><td>SF-36/RAND-36</td><td>1/116</td><td>7/12</td><td>3/3</td><td rowspan="2">4/18</td><td>10/18</td><td>8/20</td><td rowspan="2">10/22</td><td>26/40</td></tr>
<tr><td>SF-12</td><td></td><td></td><td></td><td></td><td>1/20</td><td></td></tr>
<tr><td>WHOQOL-BREF</td><td></td><td></td><td></td><td></td><td></td><td>1/20</td><td>1/22</td><td></td></tr>
<tr><td>EQ-5D</td><td></td><td></td><td></td><td>2/18</td><td></td><td>2/20</td><td></td><td></td></tr>
<tr><td>VAS</td><td></td><td></td><td></td><td></td><td></td><td>1/20</td><td></td><td></td></tr>
<tr><td>15D</td><td></td><td></td><td></td><td></td><td></td><td></td><td></td><td>1/40</td></tr>
<tr><td>NHP</td><td></td><td></td><td></td><td></td><td></td><td></td><td></td><td>2/40</td></tr>
<tr><td>COOP-WONCA</td><td></td><td></td><td></td><td></td><td></td><td></td><td>1/22</td><td></td></tr>
<tr><td colspan="9">Obesity-specific measures (n = 8 types)</td></tr>
<tr><td>IWQOL</td><td></td><td></td><td></td><td></td><td></td><td>2/20</td><td></td><td>1/40</td></tr>
<tr><td>IWQOL-Lite</td><td></td><td>1/12</td><td></td><td></td><td>2/18</td><td>6/20</td><td>3/22</td><td>5/40</td></tr>
<tr><td>QOLOD</td><td></td><td></td><td></td><td></td><td></td><td></td><td>1/22</td><td>2/40</td></tr>
<tr><td>OP</td><td></td><td>2/12</td><td></td><td></td><td>1/18</td><td></td><td></td><td>1/40</td></tr>
<tr><td>LQ</td><td></td><td>2/12</td><td></td><td></td><td></td><td></td><td></td><td></td></tr>
<tr><td>M-A QoLQII</td><td></td><td>1/12</td><td></td><td></td><td></td><td></td><td></td><td>4/40 (with BAROS)</td></tr>
<tr><td>OWLQOL</td><td></td><td></td><td></td><td></td><td></td><td></td><td></td><td rowspan="2">1/40</td></tr>
<tr><td>WRSM</td><td></td><td></td><td></td><td></td><td></td><td></td><td></td></tr>
<tr><td colspan="9">Combined generic and obesity-specific measures (n = 2 types)</td></tr>
</tbody>
</table>

SOS quality of life survey							1/40
HRQOL							1/40
**Combined obesity-specific and bariatric surgery-specific measures (*n* = 1 type)**							
BQL	1/12			2/18			
**Bariatric surgery-specific measures (*n* = 1 type)**							
BAROS	2/12			4/18			4/40 (with M-A QoLQII)
**Gastrointestinal-specific measures (*n* = 2 types)**							
GIQLI	1/12				1/20		1/40
GERD-HRQOL	1/12						
**Other disease-related measures (*n* = 5 types)**							
Health Assessment Questionnaire-Disability Index						1/22	
Knee injury and Osteoarthritis Outcome Score						1/22	
Kansas City Cardiomyopathy Questionnaire						1/22	
Minnesota Living with Heart Failure Questionnaire						1/22	
Western Ontario and McMaster Universities Osteoarthritis Index						1/22	

**Mental health-specific measures (*n* = 9 types)**		
HADS	1/18	1/20
BDI	2/18	1/20
BDI-II		2/20
CES-D	4/18	
GDS	1/18	
MADRS	1/18	
MINI	1/18	
WHO wellbeing index	1/18	
DASS-21	1/18	

Numbers represent the proportion of included studies using each type of HRQoL measure in each review.

Abbreviations: BAROS, Bariatric Analysis and Reporting Outcome System; BDI, Beck Depression Inventory; BQL, Bariatric Quality of Life; CES-D, Center for Epidemiologic Studies Depression scale; COOP-WONCA, Dartmouth Primary Care Cooperative Research Network and the World Organization of National Colleges, Academies, and Academic Associations of General Practitioners/Family Physicians; DASS-21, Depression, Anxiety, and Stress Scale-21; EQ-5D, EuroQol-5 Dimension; GDS, Geriatric Depression Scale; GERD-HRQL, Gastroesophageal Reflux Disease-Health Related Quality of Life; GIQLI, Gastrointestinal Quality of Life Index; HADS, Hospital Anxiety and Depression Scale; HRQOL, Health-Related Quality of Life questionnaire (i.e. Mathias questionnaire); HRQoL, health-related quality of life; IWQOL, Impact of Weight on Quality of Life; LQ, Laval Questionnaire; M-A QoLQII, Moorehead–Ardelt Quality of Life Questionnaire II; MADRS, Montgomery–Asberg Depression Rating Scale; MINI, Mini-International Neuropsychiatric Interview; NHP, Nottingham Health Profile; OP, Obesity-Related Problems Scale; OWLQOL, Obesity and Weight Loss Quality of Life; QOLOD, Quality of Life, Obesity and Dietetics Questionnaire; RAND-36, RAND 36-Item Health Survey; SF-12, 12-Item Short Form Survey; SF-36, 36-Item Short Form Health Survey; SOS, Swedish Obese Subjects; VAS, visual analog scale; WHO, World Health Organization; WHOQOL, World Health Organization Quality-of-Life Scale; WRSM, Weight-Related Symptom Measure.

SUPPLEMENTARY REFERENCES

1. National Institute for Health and Care Excellence. Obesity: identification, assessment and management. Clinical guideline [CG189]. July 26, 2023. Accessed April 15, 2024. https://www.nice.org.uk/guidance/cg189/chapter/Recommendations

2. Jensen MD, Ryan DH, Apovian CM, et al. 2013 AHA/ACC/TOS guideline for the management of overweight and obesity in adults: a report of the American College of Cardiology/American Heart Association Task Force on Practice Guidelines and The Obesity Society. *Circulation.* 2014;129(25 Suppl 2):S102-138. doi:10.1161/01.cir.0000437739.71477.ee

3. Mechanick JI, Apovian C, Brethauer S, et al. Clinical practice guidelines for the perioperative nutrition, metabolic, and nonsurgical support of patients undergoing bariatric procedures - 2019 update: cosponsored by American Association of Clinical Endocrinologists/American College of Endocrinology, The Obesity Society, American Society for Metabolic & Bariatric Surgery, Obesity Medicine Association, and American Society of Anesthesiologists - executive summary. *Endocr Pract.* 2019;25(12):1346-1359. doi:10.4158/gl-2019-0406

4. Grunvald E, Shah R, Hernaez R, et al. AGA clinical practice guideline on pharmacological interventions for adults with obesity. *Gastroenterology.* 2022;163(5):1198-1225. doi:10.1053/j.gastro.2022.08.045

5. Eisenberg D, Shikora SA, Aarts E, et al. 2022 American Society of Metabolic and Bariatric Surgery (ASMBS) and International Federation for the Surgery of Obesity and Metabolic Disorders (IFSO): indications for metabolic and bariatric surgery. *Obes Surg.* 2023;33(1):3-14. doi:10.1007/s11695-022-06332-1

6. Apovian CM, Aronne LJ, Bessesen DH, et al. Pharmacological management of obesity: an endocrine Society clinical practice guideline. *J Clin Endocrinol Metab.* 2015;100(2):342-362. doi:10.1210/jc.2014-3415

7. Obesity Canada. Canadian adult obesity clinical practice guidelines. Accessed April 15, 2024. https://obesitycanada.ca/guidelines/chapters/

8. Wharton S, Lau DCW, Vallis M, et al. Obesity in adults: a clinical practice guideline. *Cmaj.* 2020;192(31):E875-E891. doi:10.1503/cmaj.191707

9. Yumuk V, Tsigos C, Fried M, et al. European guidelines for obesity management in adults. *Obes Facts.* 2015;8(6):402-424. doi:10.1159/000442721

10. Scott J, Huskisson EC. Graphic representation of pain. *Pain.* 1976;2(2):175-184.

11. Fazio AF. A concurrent validational study of the NCHS General Well-Being Schedule. *Vital Health Stat 2.* 1977;(73):1-53.

12. Goldberg DP. Manual of the General Health Questionnaire. Winsor, England: NFER-Nelson Publishing; 1978

13. Davies AW, Ware JEJ. Measuring health perceptions in the health insurance experiment. Santa Monica, CA: The Rand Corporation; 1981

14. Hunt SM, McKenna SP, McEwen J, Williams J, Papp E. The Nottingham Health Profile: subjective health status and medical consultations. *Soc Sci Med A.* 1981;15(3 Pt 1):221-229. doi:10.1016/0271-7123(81)90005-5

15. Bergner M, Bobbitt RA, Carter WB, Gilson BS. The Sickness Impact Profile: development and final revision of a health status measure. *Med Care.* 1981;19(8):787-805. doi:10.1097/00005650-198108000-00001

16. Nelson EC, Landgraf JM, Hays RD, Wasson JH, Kirk JW. The functional status of patients. How can it be measured in physicians' offices? *Med Care.* 1990;28(12):1111-1126.

17. van Weel C. Measuring functional health status with the COOP/WONCA charts: a manual. Groningen, Netherlands: Northern Centre for Health Care Research; 1995

18. Hays RD, Sherbourne CD, Mazel RM. The RAND 36-Item Health Survey 1.0. *Health Econ.* 1993;2(3):217-227. doi:10.1002/hec.4730020305

19. Hays RD, Morales LS. The RAND-36 measure of health-related quality of life. *Ann Med.* 2001;33(5):350-357. doi:10.3109/07853890109002089

20. RAND. 36-Item Short Form Survey (SF-36) Scoring Instructions. Accessed March 19, 2025. https://www.rand.org/health-care/surveys_tools/mos/36-item-short-form/scoring.html

21. Ware Jr. J, Kosinski M, Keller SD. A 12-Item Short-Form Health Survey: construction of scales and preliminary tests of reliability and validity. *Med Care.* 1996;34(3):220-233. doi:10.1097/00005650-199603000-00003

22. Ware J, Kosinski M, Gandek B. SF-36 health survey: manual & interpretation guide. Lincoln, RI, USA: Quality Metric Inc.; 2000

23. Sintonen H. The 15D instrument of health-related quality of life: properties and applications. *Ann Med.* 2001;33(5):328-336. doi:10.3109/07853890109002086

24. Seiber WJ, Groessl EJ, David KM, Ganiats TG, Kaplan RM. Quality of well being self-administered (QWB-SA) scale (User's manual). San Diego, CA, USA: University of California, San Diego: Health Services Research Center; 2008

25. Gusi N, Olivares PR, Rajendram R. The EQ-5D health-related quality of life questionnaire. In: Preedy VR, Watson RR, ed. Handbook of disease burdens and quality of life measures. New York, NY, USA: Springer; 2010:87-99

26. World Health Organization. WHOQOL: measuring quality of life. Accessed April 18, 2024. https://www.who.int/tools/whoqol

27. World Health Organization. The World Health Organization Quality of Life (WHOQOL) - BREF, 2012 revision. Accessed March 18, 2025. https://iris.who.int/handle/10665/77773

28. Sullivan M, Karlsson J, Sjöström L, et al. Swedish obese subjects (SOS)--an intervention study of obesity. Baseline evaluation of health and psychosocial functioning in the first 1743 subjects examined. *Int J Obes Relat Metab Disord.* 1993;17(9):503-512.

29. Kolotkin RL, Head S, Hamilton M, Tse CK. Assessing impact of weight on quality of life. *Obes Res.* 1995;3(1):49-56. doi:10.1002/j.1550-8528.1995.tb00120.x

30. Le Pen C, Lévy E, Loos F, Banzet MN, Basdevant A. "Specific" scale compared with "generic" scale: a double measurement of the quality of life in a French community sample of obese subjects. *J Epidemiol Community Health.* 1998;52(7):445-450. doi:10.1136/jech.52.7.445

31. Mannucci E, Ricca V, Barciulli E, et al. Quality of life and overweight: the obesity related well-being (Orwell 97) questionnaire. *Addict Behav.* 1999;24(3):345-357. doi:10.1016/s0306-4603(98)00055-0

32. Butler GS, Vallis TM, Perey B, Veldhuyzen van Zanten SJ, MacDonald AS, Konok G. The Obesity Adjustment Survey: development of a scale to assess psychological adjustment to morbid obesity. *Int J Obes Relat Metab Disord.* 1999;23(5):505-511. doi:10.1038/sj.ijo.0800850

33. Kolotkin RL, Crosby RD, Kosloski KD, Williams GR. Development of a brief measure to assess quality of life in obesity. *Obes Res.* 2001;9(2):102-111. doi:10.1038/oby.2001.13

34. Kolotkin RL, Crosby RD, Williams GR. Health-related quality of life varies among obese subgroups. *Obes Res.* 2002;10(8):748-756. doi:10.1038/oby.2002.102

35. Niero M, Martin M, Finger T, et al. A new approach to multicultural item generation in the development of two obesity-specific measures: the Obesity and Weight Loss Quality of Life (OWLQOL) questionnaire and the Weight-Related Symptom Measure (WRSM). *Clin Ther.* 2002;24(4):690-700. doi:10.1016/s0149-2918(02)85144-x

36. Moorehead MK, Ardelt-Gattinger E, Lechner H, Oria HE. The validation of the Moorehead-Ardelt Quality of Life Questionnaire II. *Obes Surg.* 2003;13(5):684-692. doi:10.1381/096089203322509237

37. Ziegler O, Filipecki J, Girod I, Guillemin F. Development and validation of a French obesity-specific quality of life questionnaire: Quality of Life, Obesity and Dietetics (QOLOD) rating scale. *Diabetes Metab.* 2005;31(3 Pt 1):273-283. doi:10.1016/s1262-3636(07)70194-5

38. Therrien F, Marceau P, Turgeon N, Biron S, Richard D, Lacasse Y. The laval questionnaire: a new instrument to measure quality of life in morbid obesity. *Health Qual Life Outcomes.* 2011;9:66. doi:10.1186/1477-7525-9-66

39. Tayyem RM, Atkinson JM, Martin CR. Development and validation of a new bariatric-specific health-related quality of life instrument "bariatric and obesity-specific survey (BOSS)". *J Postgrad Med.* 2014;60(4):357-361. doi:10.4103/0022-3859.143952

40. de Vries CEE, Mou D, Poulsen L, et al. Development and validation of new BODY-Q Scales measuring expectations, eating behavior, distress, symptoms, and work life in 4004 adults from 4 countries. *Obes Surg.* 2021;31(8):3637-3645. doi:10.1007/s11695-021-05462-2

41. Klassen AF, Cano SJ, Alderman A, et al. The BODY-Q: a patient-reported outcome instrument for weight loss and body contouring treatments. *Plast Reconstr Surg Glob Open.* 2016;4(4):e679. doi:10.1097/gox.0000000000000665

42. Kolotkin RL, Ervin CM, Meincke HH, Højbjerre L, Fehnel SE. Development of a clinical trials version of the Impact of Weight on Quality of Life-Lite questionnaire (IWQOL-Lite Clinical Trials Version): results from two qualitative studies. *Clin Obes.* 2017;7(5):290-299. doi:10.1111/cob.12197

43. Kolotkin RL, Williams VSL, Ervin CM, et al. Validation of a new measure of quality of life in obesity trials: Impact of Weight on Quality of Life-Lite Clinical Trials Version. *Clin Obes.* 2019;9(3):e12310. doi:10.1111/cob.12310

44. Kolotkin RL, Williams VSL, von Huth Smith L, et al. Confirmatory psychometric evaluations of the Impact of Weight on Quality of Life-Lite Clinical Trials Version (IWQOL-Lite-CT). *Clin Obes.* 2021;11(5):e12477. doi:10.1111/cob.12477

45. Aasprang A, Våge V, Flølo TN, et al. Patient-reported quality of life with obesity - development of a new measurement scale. *Tidsskr Nor Laegeforen.* 2019;139(11). doi:10.4045/tidsskr.18.0493

46. Mathias SD, Williamson CL, Colwell HH, et al. Assessing health-related quality-of-life and health state preference in persons with obesity: a validation study. *Qual Life Res.* 1997;6(4):311-322. doi:10.1023/a:1018475108460

47. Weiner S, Sauerland S, Fein M, Blanco R, Pomhoff I, Weiner RA. The Bariatric Quality of Life index: a measure of well-being in obesity surgery patients. *Obes Surg.* 2005;15(4):538-545. doi:10.1381/0960892053723439

48. Weiner S, Sauerland S, Weiner R, Cyzewski M, Brandt J, Neugebauer E. Validation of the adapted Bariatric Quality of Life Index (BQL) in a prospective study in 446 bariatric patients as one-factor model. *Obes Facts.* 2009;2(Suppl 1):63-66. doi:10.1159/000198263

49. Zhong P, Zeng H, Huang M, Fu W, Chen Z. Efficacy and safety of once-weekly semaglutide in adults with overweight or obesity: a meta-analysis. *Endocrine.* 2022;75(3):718-724. doi:10.1007/s12020-021-02945-1

50. van den Hoek DJ, Miller CT, Fraser SF, Selig SE, Dixon JB. Does exercise training augment improvements in quality of life induced by energy restriction for obese populations? A systematic review. *Qual Life Res.* 2017;26(10):2593-2605. doi:10.1007/s11136-017-1602-9

51. Rajaie SH, Soltani S, Yazdanpanah Z, et al. Effect of exercise as adjuvant to energy-restricted diets on quality of life and depression outcomes: a meta-analysis of randomized controlled trials. *Qual Life Res.* 2022;31(11):3123-3137. doi:10.1007/s11136-022-03146-7

52. Lasikiewicz N, Myrissa K, Hoyland A, Lawton CL. Psychological benefits of weight loss following behavioural and/or dietary weight loss interventions. A systematic research review. *Appetite.* 2014;72:123-137. doi:10.1016/j.appet.2013.09.017

53. Jones RA, Lawlor ER, Birch JM, et al. The impact of adult behavioural weight management interventions on mental health: a systematic review and meta-analysis. *Obes Rev.* 2021;22(4):e13150. doi:10.1111/obr.13150

54. Vosadi E, Hashemi Fard ES, Mirakhori Z, Borjian Fard M. The impact of exercise training on psychological outcomes, body composition, and quality of life in

overweight or obese adults: a systematic review and meta-analysis of randomized controlled trials. *Biol Res Nurs.* 2025. doi:10.1177/10998004241313332

55. Hayes M, Baxter H, Müller-Nordhorn J, Hohls JK, Muckelbauer R. The longitudinal association between weight change and health-related quality of life in adults and children: a systematic review. *Obes Rev.* 2017;18(12):1398-1411. doi:10.1111/obr.12595

56. Wu F, Shi F, Fu X, Du N, Chen B, Zhou X. Laparoscopic sleeve gastrectomy versus Roux-en-Y gastric bypass for quality of life: a systematic review and meta-analysis. *Surg Obes Relat Dis.* 2020;16(11):1869-1876. doi:10.1016/j.soard.2020.06.022

57. Małczak P, Mizera M, Lee Y, et al. Quality of life after bariatric surgery-a systematic review with Bayesian network meta-analysis. *Obes Surg.* 2021;31(12):5213-5223. doi:10.1007/s11695-021-05687-1

58. Lei Y, Lei X, Chen G, et al. Update on comparison of laparoscopic sleeve gastrectomy and laparoscopic Roux-en-Y gastric bypass: a systematic review and meta-analysis of weight loss, comorbidities, and quality of life at 5 years. *BMC Surg.* 2024;24(1):219. doi:10.1186/s12893-024-02512-1

59. Hu Z, Sun J, Li R, et al. A comprehensive comparison of LRYGB and LSG in obese patients including the effects on QoL, comorbidities, weight loss, and complications: a systematic review and meta-analysis. *Obes Surg.* 2020;30(3):819-827. doi:10.1007/s11695-019-04306-4

60. García-Honores L, Caballero-Alvarado J, Bustamante-Cabrejos A, Lozano-Peralta K, Zavaleta-Corvera C. Laparoscopic sleeve gastrectomy versus laparoscopic Roux-en-Y gastric bypass for weight loss in obese patients: which is more effective? A systematic review and meta-analysis. *Arq Bras Cir Dig.* 2023;36:e1782. doi:10.1590/0102-672020230064e1782

61. Ganam S, Tang R, Sher T, Worthey A, Docimo S, Jr. Quality of life in patients undergoing revisional bariatric surgery: from sleeve gastrectomy to Roux-en-Y gastric bypass. *Obes Surg.* 2024;34(3):997-1003. doi:10.1007/s11695-024-07082-y

62. Buckell J, Mei XW, Clarke P, Aveyard P, Jebb SA. Weight loss interventions on health-related quality of life in those with moderate to severe obesity: findings from an individual patient data meta-analysis of randomized trials. *Obes Rev.* 2021;22(11):e13317. doi:10.1111/obr.13317

63. Jobanputra R, Sargeant JA, Almaqhawi A, et al. The effects of weight-lowering pharmacotherapies on physical activity, function and fitness: a systematic review and meta-analysis of randomized controlled trials. *Obes Rev.* 2023;24(4):e13553. doi:10.1111/obr.13553

64. Stewart F, Avenell A. Behavioural interventions for severe obesity before and/or after bariatric surgery: a systematic review and meta-analysis. *Obes Surg.* 2016;26(6):1203-1214. doi:10.1007/s11695-015-1873-6

65. Asiah ASS, Norhayati MN, Muhammad J, Muhamad R. Effect of yoga on anthropometry, quality of life, and lipid profile in patients with obesity and central obesity: a systematic review and meta-analysis. *Complement Ther Med.* 2023;76:102959. doi:10.1016/j.ctim.2023.102959

66. Adil MT, Jain V, Rashid F, Al-Taan O, Whitelaw D, Jambulingam P. Meta-analysis of the effect of bariatric surgery on physical function. *Br J Surg.* 2018;105(9):1107-1118. doi:10.1002/bjs.10880

67. Colquitt JL, Picot J, Loveman E, Clegg AJ. Surgery for weight loss in adults. *Cochrane Database Syst Rev.* 2014;2014(8):CD003641. doi:10.1002/14651858.CD003641.pub4

68. Colquitt JL, Picot J, Loveman E, Clegg AJ. Surgery for obesity. *Cochrane Database Syst Rev.* 2009;(2):CD003641. doi:10.1002/14651858.CD003641.pub3

69. Martín-Mariscal V. A systematic review and narrative synthesis of interventions for uncomplicated obesity: weight loss, well-being and impact on eating disorder. *Enferm Clin (Engl Ed).* 2018;28(3):212-213. doi:10.1016/j.enfcli.2017.08.004

70. Romantsova T. Effect of sibutramine (Reduxine®) on weight management and metabolic control in patients with obesity and comorbidity: a meta-analysis of observational studies. *Obes Rev.* 2020;21(Suppl 1):e13118. doi:10.1111/obr.13118

71. Vasconcelos KS, Dias JM, Araújo MC, Pinheiro AC, Moreira BS, Dias RC. Effects of a progressive resistance exercise program with high-speed component on the physical function of older women with sarcopenic obesity: a randomized controlled trial. *Braz J Phys Ther.* 2016;20(5):432-440. doi:10.1590/bjpt-rbf.2014.0174

72. Vega-Albornoz N, Navarro-Mora O, López-Espinoza MÁ. Effect of bariatric surgery on quality of life in obese patients: a global systematic review. *Revista de la Facultad de Medicina Humana.* 2023;23(4):108-116. doi:10.25176/RFMH.v23i4.5727

73. Sierżantowicz R, Ładny JR, Lewko J. Quality of life after bariatric surgery-a systematic review. *Int J Environ Res Public Health.* 2022;19(15):9078. doi:10.3390/ijerph19159078

74. Nakanishi H, Teixeira AF, Matar RH, et al. Impact on mid-term health-related quality of life after duodenal switch: a systematic review and meta-analysis. *Obes Surg.* 2023;33(3):769-779. doi:10.1007/s11695-022-06449-3

75. Gadd N, McIntosh A, Fear-Keen B, Hoult J, Maimone IR, Marshall S. Do endoscopic bariatric procedures improve postprocedural quality of life and mental health? A systematic review and meta-analysis. *Obes Surg.* 2020;30(10):4091-4100. doi:10.1007/s11695-020-04860-2

76. Abiri B, Hosseinpanah F, Banihashem S, Madinehzad SA, Valizadeh M. Mental health and quality of life in different obesity phenotypes: a systematic review. *Health Qual Life Outcomes.* 2022;20(1):63. doi:10.1186/s12955-022-01974-2

77. Baillot A, Saunders S, Brunet J, Romain AJ, Trottier A, Bernard P. A systematic review and meta-analysis of the effect of exercise on psychosocial outcomes in adults with obesity: a call for more research. *Ment Health Phys Act.* 2018;14:1-10. doi:10.1016/j.mhpa.2017.12.004

78. Raaijmakers LC, Pouwels S, Thomassen SE, Nienhuijs SW. Quality of life and bariatric surgery: a systematic review of short- and long-term results and comparison with community norms. *Eur J Clin Nutr.* 2017;71(4):441-449. doi:10.1038/ejcn.2016.198

79. Jayedi A, Soltani S, Emadi A, Zargar M-S, Najafi A. Aerobic exercise and weight loss in adults: a systematic review and dose-response meta-analysis. *JAMA Network Open.* 2024;7(12):e2452185. doi:10.1001/jamanetworkopen.2024.52185

80. Herring LY, Stevinson C, Davies MJ, et al. Changes in physical activity behaviour and physical function after bariatric surgery: a systematic review and meta-analysis. *Obes Rev.* 2016;17(3):250-261. doi:10.1111/obr.12361

81. Karlsson J, Taft C, Rydén A, Sjöström L, Sullivan M. Ten-year trends in health-related quality of life after surgical and conventional treatment for severe obesity: the SOS intervention study. *Int J Obes (Lond).* 2007;31(8):1248-1261. doi:10.1038/sj.ijo.0803573

82. Kinzl JF, Lanthaler M, Stuerz K, Aigner F. Long-term outcome after laparoscopic adjustable gastric banding for morbid obesity. *Eat Weight Disord.* 2011;16(4):e250-256. doi:10.1007/bf03327468

83. O'Brien PE, Brennan L, Laurie C, Brown W. Intensive medical weight loss or laparoscopic adjustable gastric banding in the treatment of mild to moderate obesity: long-term follow-up of a prospective randomised trial. *Obes Surg.* 2013;23(9):1345-1353. doi:10.1007/s11695-013-0990-3

84. Aarts EO, Dogan K, Koehestanie P, Aufenacker TJ, Janssen IM, Berends FJ. Long-term results after laparoscopic adjustable gastric banding: a mean fourteen year follow-up study. *Surg Obes Relat Dis.* 2014;10(4):633-640. doi:10.1016/j.soard.2014.03.019

85. Canetti L, Bachar E, Bonne O. Deterioration of mental health in bariatric surgery after 10 years despite successful weight loss. *Eur J Clin Nutr.* 2016;70(1):17-22. doi:10.1038/ejcn.2015.112

86. Herpertz S, Müller A, Burgmer R, Crosby RD, de Zwaan M, Legenbauer T. Health-related quality of life and psychological functioning 9 years after restrictive surgical treatment for obesity. *Surg Obes Relat Dis.* 2015;11(6):1361-1370. doi:10.1016/j.soard.2015.04.008

87. Aasprang A, Andersen JR, Våge V, Kolotkin R, Natvig GK. Ten-year changes in health-related quality of life after biliopancreatic diversion with duodenal switch. *Surg Obes Relat Dis.* 2016;12(8):1594-1600. doi:10.1016/j.soard.2016.04.030

88. Strain GW, Torghabeh MH, Gagner M, et al. The impact of biliopancreatic diversion with duodenal switch (BPD/DS) over 9 years. *Obes Surg.* 2017;27(3):787-794. doi:10.1007/s11695-016-2371-1

89. Nguyen NT, Kim E, Vu S, Phelan M. Ten-year outcomes of a prospective randomized trial of laparoscopic gastric bypass versus laparoscopic gastric banding. *Ann Surg.* 2018;268(1):106-113. doi:10.1097/sla.0000000000002348

90. Kolotkin RL, Kim J, Davidson LE, Crosby RD, Hunt SC, Adams TD. 12-year trajectory of health-related quality of life in gastric bypass patients versus comparison groups. *Surg Obes Relat Dis.* 2018;14(9):1359-1365. doi:10.1016/j.soard.2018.04.019

91. Rolim FFA, Cruz FS, Campos JM, Ferraz Á AB. Long-term repercussions of Roux-en-Y gastric bypass in a low-income population: assessment ten years after surgery. *Rev Col Bras Cir.* 2018;45(4):e1916. doi:10.1590/0100-6991e-20181916

92. Galli F, Cavicchioli M, Vegni E, et al. Ten years after bariatric surgery: bad quality of life promotes the need of psychological interventions. *Front Psychol.* 2018;9:2282. doi:10.3389/fpsyg.2018.02282

93. Askari A, Dai D, Taylor C, et al. Long-term outcomes and quality of life at more than 10 years after laparoscopic Roux-en-Y gastric bypass using Bariatric Analysis and Reporting Outcome System (BAROS). *Obes Surg.* 2020;30(10):3968-3973. doi:10.1007/s11695-020-04765-0

94. Neovius M, Bruze G, Jacobson P, et al. Risk of suicide and non-fatal self-harm after bariatric surgery: results from two matched cohort studies. *Lancet Diabetes Endocrinol.* 2018;6(3):197-207. doi:10.1016/s2213-8587(17)30437-0

95. Bruze G, Holmin TE, Peltonen M, et al. Associations of bariatric surgery with changes in interpersonal relationship status: results from 2 Swedish cohort studies. *JAMA Surg.* 2018;153(7):654-661. doi:10.1001/jamasurg.2018.0215

96. Legenbauer T, Müller A, de Zwaan M, Herpertz S. Body image and body avoidance nine years after bariatric surgery and conventional weight loss treatment. *Front Psychiatry.* 2019;10:945. doi:10.3389/fpsyt.2019.00945

97. Mabey JG, Kolotkin RL, Crosby RD, Crowell SE, Hunt SC, Davidson LE. Mediators of suicidality 12 years after bariatric surgery relative to a nonsurgery comparison group. *Surg Obes Relat Dis.* 2021;17(1):121-130. doi:10.1016/j.soard.2020.08.026

98. Felsenreich DM, Prager G, Kefurt R, et al. Quality of life 10 years after sleeve gastrectomy: a multicenter study. *Obes Facts.* 2019;12(2):157-166. doi:10.1159/000496296

99. Badaoui JN, Kellogg TA, Abu Dayyeh BK, et al. The outcomes of laparoscopic biliopancreatic diversion with duodenal switch on gastro-esophageal reflux disease: the Mayo Clinic experience. *Obes Surg.* 2021;31(10):4363-4370. doi:10.1007/s11695-021-05581-w

100. Biron S, Biertho L, Marceau S, Lacasse Y. Long-term follow-up of disease-specific quality of life after bariatric surgery. *Surg Obes Relat Dis.* 2018;14(5):658-664. doi:10.1016/j.soard.2018.02.009

101. Duarte MI, Bassitt DP, Azevedo OC, Waisberg J, Yamaguchi N, Pinto Junior PE. Impact on quality of life, weight loss and comorbidities: a study comparing the biliopancreatic diversion with duodenal switch and the banded Roux-en-Y gastric bypass. *Arq Gastroenterol.* 2014;51(4):320-327. doi:10.1590/s0004-28032014000400010

102. Elias K, Hedberg J, Sundbom M. Prevalence and impact of acid-related symptoms and diarrhea in patients undergoing Roux-en-Y gastric bypass, sleeve gastrectomy, and biliopancreatic diversion with duodenal switch. *Surg Obes Relat Dis.* 2020;16(4):520-527. doi:10.1016/j.soard.2019.12.020

103. Khandalavala BN, Geske J, Nirmalraj M, Sudan R. Biliopancreatic diversion revisited: health-related quality of life outcomes of biliary pancreatic with duodenal switch. *Bariatr Surg Pract Patient Care.* 2016;11(1):3-5. doi:10.1089/bari.2015.0049

104. Laurenius A, Taha O, Maleckas A, Lönroth H, Olbers T. Laparoscopic biliopancreatic diversion/duodenal switch or laparoscopic Roux-en-Y gastric bypass for super-obesity-weight loss versus side effects. *Surg Obes Relat Dis.* 2010;6(4):408-414. doi:10.1016/j.soard.2010.03.293

105. Magee CJ, Barry J, Brocklehurst J, Javed S, Macadam R, Kerrigan DD. Outcome of laparoscopic duodenal switch for morbid obesity. *Br J Surg.* 2011;98(1):79-84. doi:10.1002/bjs.7291

106. Malo FC, Marion A, Rioux A, et al. Long alimentary limb duodenal switch (LADS): an exploratory randomized trial, results at 2 years. *Obes Surg.* 2020;30(12):5047-5058. doi:10.1007/s11695-020-04968-5

107. Strain GW, Kolotkin RL, Dakin GF, et al. The effects of weight loss after bariatric surgery on health-related quality of life and depression. *Nutr Diabetes.* 2014;4(9):e132. doi:10.1038/nutd.2014.29

108. Uribarri-Gonzalez L, Nieto-Garcia L, Martis-Sueiro A, Dominguez-Muñoz JE. Impact of gastrointestinal symptoms and psychological disturbances on patients' quality of life after restrictive or malabsorptive bariatric surgery. *Gastroenterol Hepatol.* 2023;46(2):92-101. doi:10.1016/j.gastrohep.2022.02.007

109. Weiner S, Sauerland S, Weiner RA, Pomhoff I. Quality of life after bariatric surgery – Is there a difference? *Chirurgische Gastroenterologie.* 2005;21(Suppl 1):34-36. doi:10.1159/000084032

110. Ahmed HO, Ezzat RF. Quality of life of obese patients after treatment with the insertion of intra-gastric balloon versus Atkins diet in Sulaimani Governorate, Kurdistan Region, Iraq. *Ann Med Surg (Lond).* 2019;37:42-46. doi:10.1016/j.amsu.2018.11.014

111. De Castro ML, Morales MJ, Del Campo V, et al. Efficacy, safety, and tolerance of two types of intragastric balloons placed in obese subjects: a double-blind comparative study. *Obes Surg.* 2010;20(12):1642-1646. doi:10.1007/s11695-010-0128-9

112. Deliopoulou K, Konsta A, Penna S, Papakostas P, Kotzampassi K. The impact of weight loss on depression status in obese individuals subjected to intragastric balloon treatment. *Obes Surg.* 2013;23(5):669-675. doi:10.1007/s11695-012-0855-1

113. Familiari P, Costamagna G, Bléro D, et al. Transoral gastroplasty for morbid obesity: a multicenter trial with a 1-year outcome. *Gastrointest Endosc.* 2011;74(6):1248-1258. doi:10.1016/j.gie.2011.08.046

114. Fiorillo C, Quero G, Vix M, et al. 6-Month gastrointestinal quality of life (QoL) results after endoscopic sleeve gastroplasty and laparoscopic sleeve gastrectomy: a propensity score analysis. *Obes Surg.* 2020;30(5):1944-1951. doi:10.1007/s11695-020-04419-1

115. Fuller NR, Pearson S, Lau NS, et al. An intragastric balloon in the treatment of obese individuals with metabolic syndrome: a randomized controlled study. *Obesity (Silver Spring).* 2013;21(8):1561-1570. doi:10.1002/oby.20414

116. Alfredo G, Roberta M, Massimiliano C, Michele L, Nicola B, Adriano R. Long-term multiple intragastric balloon treatment--a new strategy to treat morbid obese patients refusing surgery: prospective 6-year follow-up study. *Surg Obes Relat Dis.* 2014;10(2):307-311. doi:10.1016/j.soard.2013.10.013

117. Guedes MR, Fittipaldi-Fernandez RJ, Diestel CF, Klein M. Changes in body adiposity, dietary intake, physical activity and quality of life of obese individuals submitted to intragastric balloon therapy for 6 months. *Obes Surg.* 2019;29(3):843-850. doi:10.1007/s11695-018-3609-x

118. Marinos G, Eliades C, Raman Muthusamy V, Greenway F. Weight loss and improved quality of life with a nonsurgical endoscopic treatment for obesity: clinical results from a 3- and 6-month study. *Surg Obes Relat Dis.* 2014;10(5):929-934. doi:10.1016/j.soard.2014.03.005

119. Machytka E, Gaur S, Chuttani R, et al. Elipse, the first procedureless gastric balloon for weight loss: a prospective, observational, open-label, multicenter study. *Endoscopy.* 2017;49(2):154-160. doi:10.1055/s-0042-119296

120. Moreno C, Closset J, Dugardeyn S, et al. Transoral gastroplasty is safe, feasible, and induces significant weight loss in morbidly obese patients: results of the second human pilot study. *Endoscopy.* 2008;40(5):406-413. doi:10.1055/s-2007-995748

121. Mui WL, Ng EK, Tsung BY, Lam CH, Yung MY. Impact on obesity-related illnesses and quality of life following intragastric balloon. *Obes Surg.* 2010;20(8):1128-1132. doi:10.1007/s11695-008-9766-6

122. Ponce J, Quebbemann BB, Patterson EJ. Prospective, randomized, multicenter study evaluating safety and efficacy of intragastric dual-balloon in obesity. *Surg Obes Relat Dis.* 2013;9(2):290-295. doi:10.1016/j.soard.2012.07.007

123. Norén E, Forssell H. Aspiration therapy for obesity; a safe and effective treatment. *BMC Obes.* 2016;3:56. doi:10.1186/s40608-016-0134-0

124. Tayyem RM, Obondo C, Ali A. Short-term outcome and quality of life of endoscopically placed gastric balloon and laparoscopic adjustable gastric band. *Saudi J Gastroenterol.* 2011;17(6):400-405. doi:10.4103/1319-3767.87182

125. Guedes EP, Madeira E, Mafort TT, et al. Impact of a 6-month treatment with intragastric balloon on body composition and psychopathological profile in obese individuals with metabolic syndrome. *Diabetol Metab Syndr.* 2016;8:81. doi:10.1186/s13098-016-0197-6

126. Raftopoulos I, Giannakou A. The Elipse Balloon, a swallowable gastric balloon for weight loss not requiring sedation, anesthesia or endoscopy: a pilot study with 12-month outcomes. *Surg Obes Relat Dis.* 2017;13(7):1174-1182. doi:10.1016/j.soard.2017.02.016

127. Reimão SM, da Silva MER, Nunes GC, Mestieri LHM, Dos Santos RF, de Moura EGH. Improvement of body composition and quality of life following intragastric balloon. *Obes Surg.* 2018;28(6):1806-1808. doi:10.1007/s11695-018-3209-9

128. Thompson CC, Abu Dayyeh BK, Kushner R, et al. Percutaneous gastrostomy device for the treatment of Class II and Class III obesity: results of a randomized controlled trial. *Am J Gastroenterol.* 2017;112(3):447-457. doi:10.1038/ajg.2016.500

129. Guedes EP, Madeira E, Mafort TT, et al. Impact of 6 months of treatment with intragastric balloon on body fat and quality of life in obese individuals with metabolic syndrome. *Health Qual Life Outcomes.* 2017;15(1):211. doi:10.1186/s12955-017-0790-x

130. Mehrabi F, Amiri P, Cheraghi L, Kheradmand A, Hosseinpanah F, Azizi F. Emotional states of different obesity phenotypes: a sex-specific study in a west-Asian population. *BMC Psychiatry.* 2021;21(1):124. doi:10.1186/s12888-021-03131-3

131. Kim SR, Kim HN, Song SW. Associations between mental health, quality of life, and obesity/metabolic risk phenotypes. *Metab Syndr Relat Disord.* 2020;18(7):347-352. doi:10.1089/met.2020.0028

132. Donini LM, Merola G, Poggiogalle E, et al. Disability, physical inactivity, and impaired health-related quality of life are not different in metabolically healthy vs. unhealthy obese subjects. *Nutrients.* 2016;8(12):759. doi:10.3390/nu8120759

133. Lopez-Garcia E, Guallar-Castillón P, Garcia-Esquinas E, Rodríguez-Artalejo F. Metabolically healthy obesity and health-related quality of life: a prospective cohort study. *Clin Nutr.* 2017;36(3):853-860. doi:10.1016/j.clnu.2016.04.028

134. Tsai AG, Wadden TA, Sarwer DB, et al. Metabolic syndrome and health-related quality of life in obese individuals seeking weight reduction. *Obesity (Silver Spring).* 2008;16(1):59-63. doi:10.1038/oby.2007.8

135. Phillips CM, Perry IJ. Depressive symptoms, anxiety and well-being among metabolic health obese subtypes. *Psychoneuroendocrinology.* 2015;62:47-53. doi:10.1016/j.psyneuen.2015.07.168

136. Yang Y, Herting JR, Choi J. Obesity, metabolic abnormality, and health-related quality of life by gender: a cross-sectional study in Korean adults. *Qual Life Res.* 2016;25(6):1537-1548. doi:10.1007/s11136-015-1193-2

137. Truthmann J, Mensink GBM, Bosy-Westphal A, Hapke U, Scheidt-Nave C, Schienkiewitz A. Physical health-related quality of life in relation to metabolic health and obesity among men and women in Germany. *Health Qual Life Outcomes.* 2017;15(1):122. doi:10.1186/s12955-017-0688-7

138. Delgado I, Huet L, Dexpert S, et al. Depressive symptoms in obesity: relative contribution of low-grade inflammation and metabolic health. *Psychoneuroendocrinology.* 2018;91:55-61. doi:10.1016/j.psyneuen.2018.02.030

139. Portugal-Nunes C, Reis J, Coelho A, et al. The association of metabolic dysfunction and mood across lifespan interacts with the default mode network functional connectivity. *Front Aging Neurosci.* 2021;13:618623. doi:10.3389/fnagi.2021.618623

140. Seo Y, Lee S, Ahn JS, et al. Association of metabolically healthy obesity and future depression: using national health insurance system data in Korea from 2009-2017. *Int J Environ Res Public Health.* 2020;18(1):63. doi:10.3390/ijerph18010063

141. Imbiriba L, Tess BH, Griep RH, et al. Metabolic status is not associated with job stress in individuals with obesity: the ELSA-Brasil baseline. *Int Arch Occup Environ Health.* 2021;94(4):639-646. doi:10.1007/s00420-020-01613-7

142. Yosaee S, Djafarian K, Esteghamati A, et al. Depressive symptoms among metabolically healthy and unhealthy overweight/obese individuals: a comparative study. *Med J Islam Repub Iran.* 2018;32:95. doi:10.14196/mjiri.32.95

143. Hamer M, Batty GD, Kivimaki M. Risk of future depression in people who are obese but metabolically healthy: the English longitudinal study of ageing. *Mol Psychiatry.* 2012;17(9):940-945. doi:10.1038/mp.2012.30

144. Park H, Lee K. The relationship between metabolically healthy obesity and suicidal ideation. *J Affect Disord.* 2021;292:369-374. doi:10.1016/j.jad.2021.05.101

145. Hinnouho GM, Singh-Manoux A, Gueguen A, et al. Metabolically healthy obesity and depressive symptoms: 16-year follow-up of the Gazel cohort study. *PLoS One.* 2017;12(4):e0174678. doi:10.1371/journal.pone.0174678

146. Amiri P, Jalali-Farahani S, Rezaei M, Cheraghi L, Hosseinpanah F, Azizi F. Which obesity phenotypes predict poor health-related quality of life in adult men and women? Tehran Lipid and Glucose Study. *PLoS One.* 2018;13(9):e0203028. doi:10.1371/journal.pone.0203028

147. Ul-Haq Z, Mackay DF, Fenwick E, Pell JP. Impact of metabolic comorbidity on the association between body mass index and health-related quality of life: a Scotland-wide cross-sectional study of 5,608 participants. *BMC Public Health.* 2012;12:143. doi:10.1186/1471-2458-12-143

148. Abou-Raya A, Abou-Raya S, Helmii M. Effect of exercise and dietary weight loss on symptoms and systemic inflammation in obese adults with psoriatic arthritis: randomized controlled trial. *Ann Rheum Dis.* 2014;73(Suppl 2):89. doi:10.1136/annrheumdis-2014-eular.2760

149. Christensen R, Henriksen M, Leeds AR, et al. Effect of weight maintenance on symptoms of knee osteoarthritis in obese patients: a twelve-month randomized controlled trial. *Arthritis Care Res (Hoboken).* 2015;67(5):640-650. doi:10.1002/acr.22504

150. Horwich TB, Broderick S, Chen L, et al. Relation among body mass index, exercise training, and outcomes in chronic systolic heart failure. *Am J Cardiol.* 2011;108(12):1754-1759. doi:10.1016/j.amjcard.2011.07.051

151. Imayama I, Alfano CM, Cadmus Bertram LA, et al. Effects of 12-month exercise on health-related quality of life: a randomized controlled trial. *Prev Med.* 2011;52(5):344-351. doi:10.1016/j.ypmed.2011.02.016

152. Kitzman DW, Brubaker P, Morgan T, et al. Effect of caloric restriction or aerobic exercise training on peak oxygen consumption and quality of life in obese older patients with heart failure with preserved ejection fraction: a randomized clinical trial. *JAMA.* 2016;315(1):36-46. doi:10.1001/jama.2015.17346

153. Labrunée M, Antoine D, Vergès B, Robin I, Casillas JM, Gremeaux V. Effects of a home-based rehabilitation program in obese type 2 diabetics. *Ann Phys Rehabil Med.* 2012;55(6):415-429. doi:10.1016/j.rehab.2012.06.001

154. Casilda-López J, Valenza MC, Cabrera-Martos I, Díaz-Pelegrina A, Moreno-Ramírez MP, Valenza-Demet G. Effects of a dance-based aquatic exercise program in obese

postmenopausal women with knee osteoarthritis: a randomized controlled trial. *Menopause.* 2017;24(7):768-773. doi:10.1097/gme.0000000000000841

155. Megakli T, Vlachopoulos SP, Theodorakis Y. Effects of an aerobic and resistance exercise intervention on health-related quality of life in women with obesity. *J Appl Biobehav Res.* 2016;21(2):82-106. doi:10.1111/jabr.12047

156. Megakli T, Vlachopoulos SP, Thøgersen-Ntoumani C, Theodorakis Y. Impact of aerobic and resistance exercise combination on physical self-perceptions and self-esteem in women with obesity with one-year follow-up. *Int J Sport Exerc Psychol.* 2017;15(3):236-257. doi:10.1080/1612197X.2015.1094115

157. Napoli N, Shah K, Waters DL, Sinacore DR, Qualls C, Villareal DT. Effect of weight loss, exercise, or both on cognition and quality of life in obese older adults. *Am J Clin Nutr.* 2014;100(1):189-198. doi:10.3945/ajcn.113.082883

158. Nieman DC, Custer WF, Butterworth DE, Utter AC, Henson DA. Psychological response to exercise training and/or energy restriction in obese women. *J Psychosom Res.* 2000;48(1):23-29. doi:10.1016/S0022-3999(99)00066-5

159. Plotnikoff RC, Eves N, Jung M, Sigal RJ, Padwal R, Karunamuni N. Multicomponent, home-based resistance training for obese adults with type 2 diabetes: a randomized controlled trial. *Int J Obes (Lond).* 2010;34(12):1733-1741. doi:10.1038/ijo.2010.109

160. Sarsan A, Ardiç F, Özgen M, Topuz O, Sermez Y. The effects of aerobic and resistance exercises in obese women. *Clin Rehabil.* 2006;20(9):773-782. doi:10.1177/0269215506070795

161. Sukala WR, Page R, Lonsdale C, et al. Exercise improves quality of life in indigenous polynesian peoples with type 2 diabetes and visceral obesity. *J Phys Act Health.* 2013;10(5):699-707. doi:10.1123/jpah.10.5.699

162. Svensson S, Eek F, Christiansen L, Wisén A. The effect of different exercise intensities on health related quality of life in people classified as obese. *Eur J Physiol.* 2017;19(2):104-115. doi:10.1080/21679169.2017.1296021

163. Villareal DT, Chode S, Parimi N, et al. Weight loss, exercise, or both and physical function in obese older adults. *N Engl J Med.* 2011;364(13):1218-1229. doi:10.1056/NEJMoa1008234

164. Rica RL, Carneiro RMM, Serra AJ, Rodriguez D, Pontes Junior FL, Bocalini DS. Effects of water-based exercise in obese older women: impact of short-term follow-up study on anthropometric, functional fitness and quality of life parameters. *Geriatr Gerontol Int.* 2013;13(1):209-214. doi:10.1111/j.1447-0594.2012.00889.x

165. Baillot A, Vibarel-Rebot N, Amiot V, Emy P, Collomp K. Effects of an 8-week aerobic exercise training on saliva steroid hormones, physical capacity, and quality of life in diabetic obese men. *Horm Metab Res.* 2012;44(2):146-151. doi:10.1055/s-0031-1297262

166. del Rey-Moya LM, Castilla-Álvarez C, Pichiule-Castañeda M, et al. Effect of a group intervention in the primary healthcare setting on continuing adherence to physical exercise routines in obese women. *J Clin Nurs.* 2013;22(15-16):2114-2121. doi:10.1111/jocn.12091

167. Lucha-López MO, Lucha-López AC, Vidal-Peracho C, et al. Impact of supervised physiotherapeutic exercises for obese adults with diabetes mellitus type 2. *J Phys Ther Sci.* 2012;24(12):1299-1305. doi:10.1589/jpts.24.1299

168. Muller-Pinget S, Carrard I, Ybarra J, Golay A. Dance therapy improves self-body image among obese patients. *Patient Educ Couns.* 2012;89(3):525-528. doi:10.1016/j.pec.2012.07.008

169. Wouters EJM, Van Nunen AMA, Geenen R, Kolotkin RL, Vingerhoets AJJM. Effects of aquajogging in obese adults: a pilot study. *J Obes.* 2010;2010(1):231074. doi:10.1155/2010/231074

170. Grans R, Warth CF, Farah JF, Bassitt DP. Quality of life and prevalence of osteoarticular pain in patients submitted to bariatric surgery. *Einstein (Sao Paulo).* 2012;10(4):415-421. doi:10.1590/s1679-45082012000400004

171. Klingemann J, Pataky Z, Iliescu I, Golay A. Relationship between quality of life and weight loss 1 year after gastric bypass. *Dig Surg.* 2009;26(5):430-433. doi:10.1159/000237746

172. Lier HO, Biringer E, Hove O, Stubhaug B, Tangen T. Quality of life among patients undergoing bariatric surgery: associations with mental health - a 1 year follow-up study of bariatric surgery patients. *Health Qual Life Outcomes.* 2011;9:79. doi:10.1186/1477-7525-9-79

173. Martínez Y, Ruiz-López MD, Giménez R, Pérez de la Cruz AJ, Orduña R. Does bariatric surgery improve the patient's quality of life? *Nutr Hosp.* 2010;25(6):925-930.

174. Freys SM, Tigges H, Heimbucher J, Fuchs KH, Fein M, Thiede A. Quality of life following laparoscopic gastric banding in patients with morbid obesity. *J Gastrointest Surg.* 2001;5(4):401-407. doi:10.1016/s1091-255x(01)80069-x

175. van Hout GC, Fortuin FA, Pelle AJ, Blokland-Koomen ME, van Heck GL. Health-related quality of life following vertical banded gastroplasty. *Surg Endosc.* 2009;23(3):550-556. doi:10.1007/s00464-008-9860-9

176. Aarts F, Hinnen C, Gerdes VEA, Brandjes DPM, Geenen R. The significance of attachment representations for quality of life one year following gastric bypass surgery: a longitudinal analysis. *Bariatr Surg Pract Patient Care.* 2014;9(3):113-118. doi:10.1089/bari.2014.0022

177. Aasprang A, Andersen JR, Våge V, Kolotkin RL, Natvig GK. Five-year changes in health-related quality of life after biliopancreatic diversion with duodenal switch. *Obes Surg.* 2013;23(10):1662-1668. doi:10.1007/s11695-013-0994-z

178. Adami GF, Ramberti G, Weiss A, Carlini F, Murelli F, Scopinaro N. Quality of life in obese subjects following biliopancreatic diversion. *Behav Med.* 2005;31(2):53-60. doi:10.3200/bmed.31.2.53-62

179. Adams TD, Pendleton RC, Strong MB, et al. Health outcomes of gastric bypass patients compared to nonsurgical, nonintervened severely obese. *Obesity (Silver Spring).* 2010;18(1):121-130. doi:10.1038/oby.2009.178

180. Adams TD, Davidson LE, Litwin SE, et al. Health benefits of gastric bypass surgery after 6 years. *JAMA.* 2012;308(11):1122-1131. doi:10.1001/2012.jama.11164

181. Ahroni JH, Montgomery KF, Watkins BM. Laparoscopic adjustable gastric banding: weight loss, co-morbidities, medication usage and quality of life at one year. *Obes Surg.* 2005;15(5):641-647. doi:10.1381/0960892053923716

182. Boan J, Kolotkin RL, Westman EC, McMahon RL, Grant JP. Binge eating, quality of life and physical activity improve after Roux-en-Y gastric bypass for morbid obesity. *Obes Surg.* 2004;14(3):341-348. doi:10.1381/096089204322917864

183. Brunault P, Frammery J, Couet C, et al. Predictors of changes in physical, psychosocial, sexual quality of life, and comfort with food after obesity surgery: a 12-month follow-up study. *Qual Life Res.* 2015;24(2):493-501. doi:10.1007/s11136-014-0775-8

184. Brunault P, Jacobi D, Léger J, et al. Observations regarding 'quality of life' and 'comfort with food' after bariatric surgery: comparison between laparoscopic adjustable gastric banding and sleeve gastrectomy. *Obes Surg.* 2011;21(8):1225-1231. doi:10.1007/s11695-011-0411-4

185. Busetto L, Mozzi E, Schettino AM, et al. Three years durability of the improvements in health-related quality of life observed after gastric banding. *Surg Obes Relat Dis.* 2015;11(1):110-117. doi:10.1016/j.soard.2014.04.016

186. Charalampakis V, Bertsias G, Lamprou V, de Bree E, Romanos J, Melissas J. Quality of life before and after laparoscopic sleeve gastrectomy. A prospective cohort study. *Surg Obes Relat Dis.* 2015;11(1):70-76. doi:10.1016/j.soard.2014.04.024

187. Efthymiou V, Hyphantis T, Karaivazoglou K, et al. The effect of bariatric surgery on patient HRQOL and sexual health during a 1-year postoperative period. *Obes Surg.* 2015;25(2):310-318. doi:10.1007/s11695-014-1384-x

188. Fezzi M, Kolotkin RL, Nedelcu M, et al. Improvement in quality of life after laparoscopic sleeve gastrectomy. *Obes Surg.* 2011;21(8):1161-1167. doi:10.1007/s11695-011-0361-x

189. Hansen NB, Gudex C, Støving RK. Improvement in health-related quality of life following Roux-en-Y gastric bypass. *Dan Med J.* 2014;61(7):A4870.

190. Helmiö M, Salminen P, Sintonen H, Ovaska J, Victorzon M. A 5-year prospective quality of life analysis following laparoscopic adjustable gastric banding for morbid obesity. *Obes Surg.* 2011;21(10):1585-1591. doi:10.1007/s11695-011-0425-y

191. Julia C, Ciangura C, Capuron L, et al. Quality of life after Roux-en-Y gastric bypass and changes in body mass index and obesity-related comorbidities. *Diabetes Metab.* 2013;39(2):148-154. doi:10.1016/j.diabet.2012.10.008

192. Karlsen TI, Lund RS, Røislien J, et al. Health related quality of life after gastric bypass or intensive lifestyle intervention: a controlled clinical study. *Health Qual Life Outcomes.* 2013;11:17. doi:10.1186/1477-7525-11-17

193. Kolotkin RL, Crosby RD, Gress RE, Hunt SC, Adams TD. Two-year changes in health-related quality of life in gastric bypass patients compared with severely obese controls. *Surg Obes Relat Dis.* 2009;5(2):250-256. doi:10.1016/j.soard.2009.01.009

194. Kolotkin RL, Davidson LE, Crosby RD, Hunt SC, Adams TD. Six-year changes in health-related quality of life in gastric bypass patients versus obese comparison groups. *Surg Obes Relat Dis.* 2012;8(5):625-633. doi:10.1016/j.soard.2012.01.011

195. Lee WJ, Yu PJ, Wang W, Lin CM, Wei PL, Huang MT. Gastrointestinal quality of life following laparoscopic vertical banded gastroplasty. *Obes Surg.* 2002;12(6):819-824; discussion 825. doi:10.1381/096089202320995628

196. Major P, Matłok M, Pędziwiatr M, et al. Quality of life after bariatric surgery. *Obes Surg.* 2015;25(9):1703-1710. doi:10.1007/s11695-015-1601-2

197. Mar J, Karlsson J, Arrospide A, Mar B, Martínez de Aragón G, Martinez-Blazquez C. Two-year changes in generic and obesity-specific quality of life after gastric bypass. *Eat Weight Disord.* 2013;18(3):305-310. doi:10.1007/s40519-013-0039-6

198. Mathus-Vliegen EM, de Wit LT. Health-related quality of life after gastric banding. *Br J Surg.* 2007;94(4):457-465. doi:10.1002/bjs.5607

199. Nadalini L, Zenti MG, Masotto L, et al. Improved quality of life after bariatric surgery in morbidly obese patients. Interdisciplinary group of bariatric surgery of Verona (G.I.C.O.V.). *G Chir.* 2014;35(7-8):161-164.

200. O'Brien PE, Dixon JB, Laurie C, Anderson M. A prospective randomized trial of placement of the laparoscopic adjustable gastric band: comparison of the perigastric and pars flaccida pathways. *Obes Surg.* 2005;15(6):820-826. doi:10.1381/0960892054222858

201. Omotosho P, Mor A, Shantavasinkul PC, Corsino L, Torquati A. Gastric bypass significantly improves quality of life in morbidly obese patients with type 2 diabetes. *Surg Endosc.* 2016;30(7):2857-2864. doi:10.1007/s00464-015-4568-0

202. Peterli R, Borbély Y, Kern B, et al. Early results of the Swiss Multicentre Bypass or Sleeve Study (SM-BOSS): a prospective randomized trial comparing laparoscopic sleeve gastrectomy and Roux-en-Y gastric bypass. *Ann Surg.* 2013;258(5):690-694; discussion 695. doi:10.1097/SLA.0b013e3182a67426

203. Pilone V, Mozzi E, Schettino AM, et al. Improvement in health-related quality of life in first year after laparoscopic adjustable gastric banding. *Surg Obes Relat Dis.* 2012;8(3):260-268. doi:10.1016/j.soard.2011.12.012

204. Risstad H, Søvik TT, Engström M, et al. Five-year outcomes after laparoscopic gastric bypass and laparoscopic duodenal switch in patients with body mass index of 50 to 60: a randomized clinical trial. *JAMA Surg.* 2015;150(4):352-361. doi:10.1001/jamasurg.2014.3579

205. Suter M, Donadini A, Romy S, Demartines N, Giusti V. Laparoscopic Roux-en-Y gastric bypass: significant long-term weight loss, improvement of obesity-related comorbidities and quality of life. *Ann Surg.* 2011;254(2):267-273. doi:10.1097/SLA.0b013e3182263b66

206. Warkentin LM, Majumdar SR, Johnson JA, et al. Weight loss required by the severely obese to achieve clinically important differences in health-related quality of life: two-year prospective cohort study. *BMC Med.* 2014;12:175. doi:10.1186/s12916-014-0175-5

207. White MA, Kalarchian MA, Levine MD, Masheb RM, Marcus MD, Grilo CM. Prognostic significance of depressive symptoms on weight loss and psychosocial outcomes following gastric bypass surgery: a prospective 24-month follow-up study. *Obes Surg.* 2015;25(10):1909-1916. doi:10.1007/s11695-015-1631-9

208. Zijlstra H, Larsen JK, Wouters EJM, van Ramshorst B, Geenen R. The long-term course of quality of life and the prediction of weight outcome after laparoscopic adjustable gastric banding: a prospective study. *Bariatr Surg Pract Patient Care.* 2013;8(1):18-22. doi:10.1089/bari.2013.9998

209. Villareal DT, Aguirre L, Gurney AB, et al. Aerobic or resistance exercise, or both, in dieting obese older adults. *N Engl J Med.* 2017;376(20):1943-1955. doi:10.1056/NEJMoa1616338
